# Global LC/MS Metabolomics Profiling of Calcium Stressed and Immunosuppressant Drug Treated *Saccharomyces cerevisiae*

**DOI:** 10.3390/metabo3041102

**Published:** 2013-12-06

**Authors:** Stefan Jenkins, Steven M. Fischer, Lily Chen, Theodore R. Sana

**Affiliations:** 1 Life Sciences Division, Lawrence Berkeley National Laboratory, Berkeley, CA 94720, USA; 2 Agilent Technologies, Life Sciences, Diagnostics and Applied Markets, Santa Clara, CA 95051, USA; E-Mails: theodore_sana@agilent.com (S.M.F.); sfischer@agilent.com (T.R.S.); 3 Biology Department, San Francisco State University, San Francisco, CA 94132, USA; E-Mail: lilychen@sfsu.edu

**Keywords:** untargeted metabolomics, *Saccharomyces cerevisiae*, yeast, calcium, calcineurin, FK506, Cyclosporin A, stress response

## Abstract

Previous studies have shown that calcium stressed *Saccharomyces cerevisiae*, challenged with immunosuppressant drugs FK506 and Cyclosporin A, responds with comprehensive gene expression changes and attenuation of the generalized calcium stress response. Here, we describe a global metabolomics workflow for investigating the utility of tracking corresponding phenotypic changes. This was achieved by efficiently analyzing relative abundance differences between intracellular metabolite pools from wild-type and calcium stressed cultures, with and without prior immunosuppressant drugs exposure. We used pathway database content from WikiPathways and YeastCyc to facilitate the projection of our metabolomics profiling results onto biological pathways. A key challenge was to increase the coverage of the detected metabolites. This was achieved by applying both reverse phase (RP) and aqueous normal phase (ANP) chromatographic separations, as well as electrospray ionization (ESI) and atmospheric pressure chemical ionization (APCI) sources for detection in both ion polarities. Unsupervised principle component analysis (PCA) and ANOVA results revealed differentiation between wild-type controls, calcium stressed and immunosuppressant/calcium challenged cells. Untargeted data mining resulted in 247 differentially expressed, annotated metabolites, across at least one pair of conditions. A separate, targeted data mining strategy identified 187 differential, annotated metabolites. All annotated metabolites were subsequently mapped onto curated pathways from YeastCyc and WikiPathways for interactive pathway analysis and visualization. Dozens of pathways showed differential responses to stress conditions based on one or more matches to the list of annotated metabolites or to metabolites that had been identified further by MS/MS. The purine salvage, pantothenate and sulfur amino acid pathways were flagged as being enriched, which is consistent with previously published literature for transcriptomics analysis. Thus, broad discovery-based data mining combined with targeted pathway projections can be an important asset for rapidly distilling, testing and evaluating a large amount of information for further investigation.

## 1. Introduction

The feasibility of using a global metabolite profiling workflow to begin assessing the intrinsic response of *Saccharomyces cerevisiae* to calcium stress and immunosuppressant drug challenge was investigated.

Calcium is a critical second messenger and is required in many enzymatic reactions. In order to maintain homeostasis during sudden fluctuations in extracellular calcium concentrations, *S. cerevisiae* employs an environmental stress response (ESR) that results in profound changes to its intracellular chemistry, as well as gene expression [1,2,3]. Two-hybrid and microarray experiments have previously shown that the *S. cerevisiae* response to calcium exposure is immediate with rapid changes in gene transcription [3,4,5]. Calcineurin, a Ca^2+^/calmodulin-dependent protein phosphatase, is a critical component of calcium regulated signaling in *S. cerevisiae* [6,7]. Calcium homeostasis and calcineurin function are critical to eukaryotic health, with disruption in humans being linked to multiple diseases and pathologies, such as Down’s syndrome and Alzheimer’s [8,9,10,11].

Since the immunosuppressant drugs, FK506 and Cyclosporin A (CsA), are immunophilin ligands that specifically inhibit calcineurin, with immediate effects on transcription [6], they are valuable clinical drugs, as well as important experimental tools for probing the actions of the calcineurin-mediated response to environmental stress. Kadafar and Cyert [7] have reported that calcium challenges in *S. cerevisiae* result in over one hundred differential genes of which approximately 90% showed a significant reduction in expression when subsequently treated with FK506. The metabolomics profile in response to calcium stress and immunosuppressant drugs, however, has not been reported.

The metabolome reflects the small molecule products of cellular metabolic and regulatory processes and encompasses a broad range of physico-chemical properties [12]. This presents a significant challenge for comprehensive metabolite profiling, which, on a global scale, requires different analytical approaches [13,14,15,16,17,18,19,20]. We therefore addressed the separation issues for different classes of metabolites [21,22] by using dual chromatographic separation, as well as multiple mass spectrometer (MS) detection modes.

Both discovery-based targeted and untargeted data mining approaches were used to distill raw datasets. The targeted data mining approach used an annotated list of entities based on compounds known to be associated with the *S. cerevisiae* metabolome, while the purpose of our untargeted data mining approach was to find correlations between conditions based on a list of entities after differential analysis and annotation via matching to the METLIN [23] accurate mass database. Lastly, in order to provide preliminary tracking of metabolites that did not match METLIN, our untargeted data mining approach also employed a molecular formula generation algorithm for calculating the best empirical formula based on accurate mass and isotope information. Systematic analysis and visualization of the experimental results was accomplished via seamless integration of multiple wizards and modules in the software. The identity of a subset of differential metabolites was confirmed through accurate mass matching and/or by MS/MS spectral library identification. The visualization of metabolite relative abundances by ontology mapping onto biochemical pathways was a key component of our global, untargeted workflow and provided a framework for rapidly inferring the response of *S. cerevisiae* to calcium and immunosuppressant drug treatment. Moreover, our results suggest that in future experiments, both calcium and immunosuppressant drug concentrations, as well as dynamics (time) can be varied, which will be important for teasing out the correlation between transcripts and metabolites in response to stress in more detail. The results of this study could have important implications for using global profiling as a routine tool for understanding the results of pathway-focused metabolomics experiments.

## 2. Materials and Methods

Data for five separate LC/MS “analytical conditions” were collected: (1) reverse phase electrospray ionization in positive ion polarity: RP-ESI+; (2) RP-ESI−; (3) aqueous normal phase electrospray ionization in positive ion polarity: ANP-ESI+; (4) ANP-ESI−; and (5) reverse phase atmospheric chemical ionization positive ion polarity; RP-APCI+.

### 2.1. Yeast Culture

The culture methodology employed was based on that reported by Stathopoulos and Cyert with slight modification [1]; cultures were vehicle control or drug treated for 1 h, with select cultures subsequently challenged by calcium exposure for 15 min. *S. cerevisiae* strain BJ5459 (generously supplied by Dr. Martha Cyert of Stanford University) was cultured in YPD media (2% peptone, 2% dextrose, 1% yeast extract; MP Biomedical, Santa Ana calcium) at 30 °C to an OD_600_ of 0.8. Cultures were then treated with the vehicle control only (wild-type and calcium only (calcium) treatments) or with an immunosuppressant drug (FK506 or CsA), both added to a final concentration of 5 µg/mL. After 1 h, the calcium, FK506 and CsA cultures were adjusted to 0.2 M CaCl_2_ (and 4 µg/mL of FK506 or CsA) for 15 min at 30 °C, after which all cultures were centrifuged, rinsed and quenched. See Supplemental Note N1 of the Supplementary Materials for the detailed culturing methodology.

### 2.2. Metabolite Extraction

Extraction using a chloroform:methanol:buffer system was described by de Koning and van Dam, with efficacy confirmed by Villas-Boas *et al*. [20,24]. In order to prepare extracts directly for MS analysis, water was used instead of PIPES/EDTA buffer. The separation and recovery of nonpolar metabolites in the chloroform phase was used for atmospheric pressure chemical ionization (APCI) MS analysis, as one of the multiple steps taken to broaden the analytical scope of our experiments.

For each “treatment condition” (wild-type, calcium, FK506 and CsA), 9 biological replicates were prepared: 5 mg of dry yeast was weighed out into chilled 2 mL tubes to which was added a 5-mm stainless steel ball bearing. 9-anthracene carboxylic acid and 1-napthylamine external standards were added in 1.1 mL of 5:3:3 chloroform:methanol:water extraction solvent to track extraction efficiency at a final concentration of 5 μg/mL. Metabolite extraction was done using a mixer mill and subsequent biphasic separation, resulting in polar and non-polar phase samples. See Supplemental Note N2 in the Supplementary Materials for the detailed extraction methodology.

Extraction controls were prepared by following the same protocol without adding any dried yeast to the extraction tubes. By completely processing these control samples, we were able to account for any MS signal contribution from the materials and reagents used.

### 2.3. Trace Protein and Cell Debris Removal

Trace proteins and/or carry-over cell debris in the polar phase samples were removed by filtration of all samples through 0.2 µm microfiltration tubes, followed by 10 kDa, Nanosep MF Centrifugal Devices (Pall Corporation, Port Washington, NY, USA), according to the manufacturer instructions. All samples were then lyophilized at −60 °C and stored at −80 °C.

### 2.4. Chromatography

Reverse phase (RP) chromatography was performed using an Agilent ZORBAX C18 SB-Aq (Agilent Technologies, Santa Clara, CA, USA) 2.1 mm × 50 mm, 1.8 µm particle column. An Agilent ZORBAX C-8 (Agilent Technologies, Santa Clara, CA, USA), 2.1 mm × 30 mm, 3.5-µm particle guard column was placed in series in front of the analytical column. An Agilent 1200 SL Series HPLC system (Agilent Technologies, Santa Clara, CA, USA) with a binary pump and degasser, thermostated well plate autosampler and thermostated column compartment was used. The autosampler temperature was 4 °C, the injection volume, 5 µL, column temperature, 60 °C, and the flow rate, 0.6 mL/min. A 2%–98% linear gradient of solvent A (0.2% acetic acid in water) to B (0.2% acetic acid in methanol (Honeywell, Morristown, NJ, USA)), was employed over 16 min followed by a solvent B hold of 2 min and a 5 min post-time for both positive and negative ion polarity analysis.

Aqueous normal phase (ANP) chromatography was done using a novel 4-μm particle size silica hydride stationary phase material column in 2.1 mm × 150 mm dimensions (MicroSolv, Eatontown, NJ, USA). The injection volume was 2 μL and column thermostat temperature, 60 °C. ANP-ESI+ chromatography was done using a 3%–80% linear gradient of solvent A (0.1% formic acid in 1:1 water:isopropanol (Honeywell, Morristown, NJ, USA)) to B (0.1% formic acid in 97:3 acetonitrile (Honeywell, Morristown, NJ, USA):water) employed over 15 min followed by a 5 min post-time. ANP-ESI− was done using a 1%–80% linear gradient of solvent A (0.025% formic acid and 5 μM EDTA in 1:1 water:isopropanol (Honeywell, Morristown, NJ, USA)) to B (5 mM ammonium formate with 5 μM EDTA in 9:1 acetonitrile (Honeywell, Morristown, NJ, USA):water) employed over 15 min followed by a 5 min post-time.

### 2.5. MS and MS/MS

An Agilent 6530 Accurate-Mass Quadrupole-Time of Flight (Q-TOF) mass spectrometer (MS) (Agilent Technologies, Santa Clara, CA, USA) was operated in ESI+ and ESI− (no switching) and in APCI+ modes. Dynamic mass axis calibration was achieved by continuous infusion of a reference mass solution (121.050873 and 922.009798 for positive polarity and 119.036320 and 966.000725 for negative polarity). Scanning conditions were as follows: drying gas temperature of 325 °C and flow rate of 10 L/min for ESI and 5 L/min for APCI+; vaporizer temperature of 350 °C; nebulizer pressure of 45 psi; capillary voltage 4,000 V in ESI+ and ESI−, 3,500 V in APCI+ with a corona current of 4 µA. See Supplementary Table S1 for additional MS conditions.

A list of precursor ions from the custom Personal Compound Database (PCD) was used to build an auto MS/MS method. The list of precursor ions was cross-checked for MS/MS spectral data for over 2,000 standards in the METLIN PCDL (Personal Compound Database and Library) of metabolites. This ensured that acquired MS/MS spectral data from our samples could be queried against the data from standards in the PCDL. Data was acquired at 3 collision energies: 10, 20 and 40 eV. A target MS/MS method was also built from untargeted data mining results directed at previously observed retention times and masses. Samples were re-run with the following MS/MS parameters: nebulizer pressure 35 psi; drying gas flow rate and temperature 9 L/min and 325 °C; capillary voltage 4,000 V in positive polarity, 3,500 V in negative polarity; collision energy 10, 20 or 40 eV; fragmentor 200 V. See Supplementary Table S1 for additional MS/MS conditions.

### 2.6. Data Analysis

#### 2.6.1. Personal Compound Database (PCD) and Library (PCDL)

All features found by untargeted data mining were queried against the METLIN Personal Compound Database (PCD), an editable database containing approximately 25,000 metabolites, providing one dimensional compound identification by accurate mass.

A second “targeted” PCD was created that was based on a list of 843 metabolites and their corresponding empirical formulas present in *S. cerevisiae* pathways from KEGG (Kyoto Encyclopedia of Genes and Genomes) and from YeastCyc [25]. This database was used for targeted mining of raw data in terms of the known *S. cerevisiae* metabolome. Finally, a PCDL containing MS/MS spectral information for over 2,000 metabolite standards, acquired at 10, 20 and 40 eV, was used to match against acquired sample fragmentation spectra contained in the library.

#### 2.6.2. Feature Extraction

For untargeted data mining, the raw data processing strategy was as previously described [19]. Briefly, the molecular feature extractor (MFE) algorithm in Agilent MassHunter Qualitative Analysis B.05.00 (Agilent Technologies, Santa Clara, CA, USA) was used to find compounds by locating covariant ions. In addition to using chromatographic peak information, the algorithm uses mass accuracy to group related ions by charge-state envelope, isotopic distribution and/or the presence of adducts and dimers. It assigns multiple species (ions) that are related to the same neutral molecule to a single compound, which is referred to as a feature. Using this approach, MFE located multiple compounds within a single chromatographic peak. The resulting refined feature list is saved to the original data file and available for downstream processing. Extraction controls and blank injection data files were generated and used to compile exclusion lists to ensure that final feature lists contained no signal contribution from the extraction process or instrumentation.

The untargeted data mining approach provided a preliminary step towards finding metabolites and expansion of the *S. cerevisiae* metabolome. Differential features derived from MFE results were first queried against METLIN content by accurate mass using the ID Browser function in Mass Profiler Professional (Agilent Technologies, Santa Clara, CA, USA), intended to provide an initial annotation for features that may be metabolites present in METLIN, but previously unknown to the *S. cerevisiae* metabolome. Assignment of a putative molecular formula to un-annotated features was intended as a preliminary step for the tracking of compounds that could be unknown metabolites. Targeted data mining used the list of annotated formulas from the custom, *S. cerevisiae*-specific PCD, for finding features using the Find by Formula (FbF) algorithm in MassHunter Qualitative Analysis B.05.00. The results were summarized as a detected entity based on Extracted Ion Count (EIC) chromatograms, set to a window of 5 ppm mass error. The resulting entity lists were saved to the original data file, exported for subsequent alignment, statistical analysis and visualization purposes.

#### 2.6.3. Baselining, Alignment, Filtering and Statistical Analysis

The results for both untargeted and targeted data mining were exported to Mass Profiler Professional (MPP), a multivariate data analysis and visualization software package that enabled alignment and binning for differential and statistical analysis, as well as pathway analysis. Baselining the entity abundances was performed to the median of all samples, where the abundance for each compound was log (base 2) normalized. The total number of features across the experiment was calculated for both untargeted and targeted data mining results. Initial tolerances were applied, where a particular feature was required to be present within 0.3 min retention time and 5 ppm mass error windows. For untargeted data mining, a secondary filtering step included a requirement for a feature to be present in at least 6 of 9 biological replicates. A similar filter was set for targeted data mining, where a feature needed to be present in at least 4 of 9 biological replicates and for both data mining approaches in at least 1 of the 4 treatment conditions. Subsequent determination of differential and statistical (*p* < 0.05) significance reduced the feature list down to a final list of target compounds that were differentially expressed pairwise between different treatment conditions.

#### 2.6.4. Pathway Analysis

Projection of pathway results was done with the Pathway Analysis module in MPP. This module supports data analysis for curated pathways from the YeastCyc collection. YeastCyc is a biochemical pathway database for *S. cerevisiae* and was created computationally by predicting the metabolic pathways of an organism by comparing the annotated genome to a reference database of manually curated, experimentally determined metabolic pathways [25].

We imported all documented pathways for *S. cerevisiae*, querying the list of pathway associated compounds against our sample derived lists of annotated compounds and looked for significant overlap. The identified significant pathways were graphically rendered with details, such as compound name and log2 normalized abundance ratios represented by a heat strip.

## 3. Results and Discussion

### 3.1. Untargeted Data Mining

RP chromatography showed extensive co-elution of compounds within a very busy void volume region, consistent with the limited retention of highly polar metabolites. However, the hydrophilic silica hydride stationary phase used for ANP chromatography was effective in separating these early eluting compounds, reducing co-elution and ion suppression.

**Figure 1:**
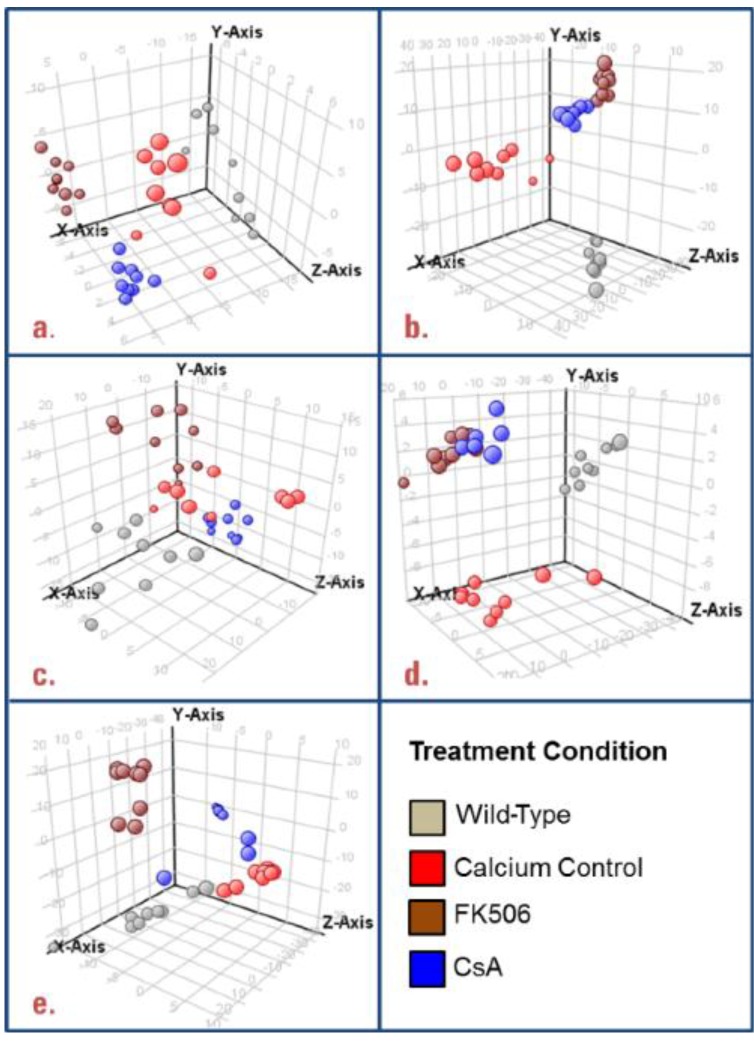
PCA plots for unannotated untargeted data mining features generated using data alignment, visualization and statistical analysis software. Clustering within each treatment condition (*n* = 9), as well as separation between conditions was observed in each of the analytical conditions ((**a**) reverse phase (RP)- electrospray ionization (ESI)+, (**b**) RP-ESI−, (**c**) aqueous normal phase (ANP)-ESI+, (**d**) ANP-ESI−, (**e**) RP-APCI+). CsA, Cyclosporin A.

Unsupervised PCA analyses (Figure 1a–e) revealed that, overall, we observed clustering of biological replicates and separation between the various conditions. We observed 204 non-redundant, annotated matches to the METLIN database that were significantly differential (*p* < 0.05) between one or more pairwise conditions (Supplementary Table S2). An additional 188 features were also differential at *p* < 0.05, with a putative molecular formula assigned based on a molecular formula calculation, each formula having a quality match score of at least 80 out of 100 (Supplementary Table S3). These preliminary untargeted data mining results were intended to provide a first step towards: (1) correlating known metabolites to the *S. cerevisiae* metabolome; and (2) providing putative molecular formulas for metabolites that did not match a METLIN database entry. The technical system and workflow described could be used to provide a qualitative investigation of these results.

### 3.2. Targeted Data Mining

**Figure 2:**
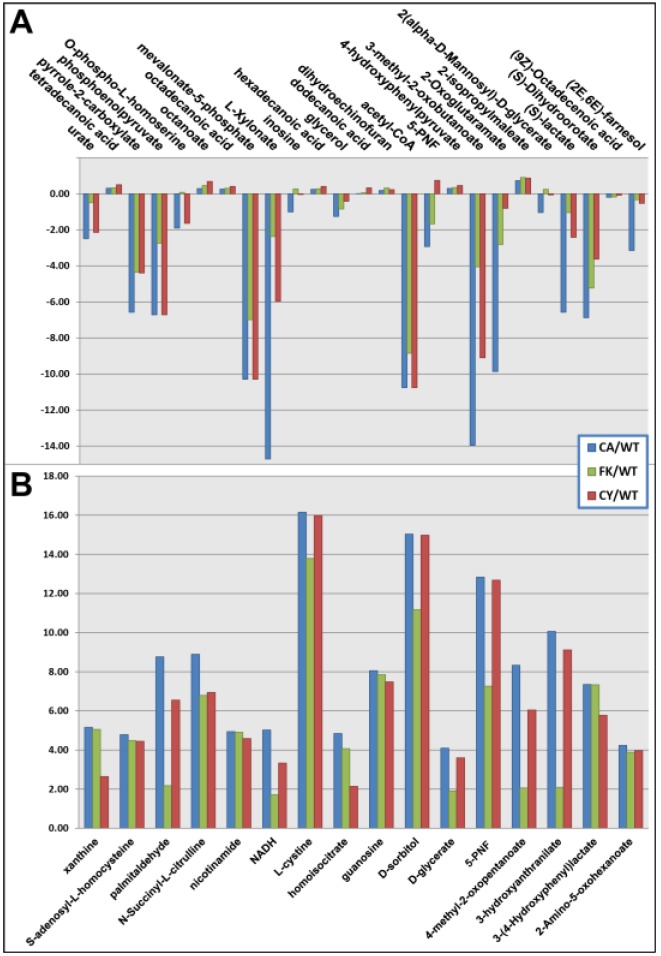
Annotated features from targeted mining results detected at highest and lowest levels in wild-type and calcium treated datasets. (**A**) Twenty-five annotated features detected at the highest level in wild-type and the lowest level in calcium treated datasets. (**B**) Sixteen annotated features detected at the highest level in calcium treated and the lowest level in wild-type datasets. CA/WT, calcium to wild-type signal ratio; CY/WT, CsA to wild-type signal ratio; FK/WT, FK506 to wild-type signal ratio; 5-PNF, 5-phosphoribosyl-N-formylglycineamidine.

Targeted data mining of the RP-ESI+ and RP-ESI− datasets revealed 186 differential features at *p* < 0.05, a subset of which were subsequently confirmed by MS/MS. (Supplementary Table S5). Of particular interest were the two subsets of entities that showed the highest and lowest abundances in wild-type and calcium treated datasets; 25 were highest in wild-type cells and lowest in calcium treated; 16 were found to be highest in calcium treated *vs.* wild-type (Figure 2). This pattern is consistent with previously reported gene expression programs associated with the calcineurin-mediated *S. cerevisiae* ESR [3,11]. Specifically, the effect of calcium treatment, whether to increase or decrease detected metabolite abundance, was attenuated by prior exposure to FK506 or CsA, but not to the baseline levels established in the wild-type. Hexadecanoic and octadecanoic fatty acids have previously been reported in a group of 20 metabolites differentially responsive to the effects of ethanol accumulation [26]. A total of 10 of those metabolites were annotated to differential features in our results (Supplementary Table S4). The correlation between ethanol exposure and the calcineurin-mediated ESR has been confirmed with the calcineurin-dependent response element (CDRE) dependent expression and Crz1p nuclear localization reported as stimulated by ethanol exposure, in addition to *CRZ1* upregulation conferring increased viability in high ethanol conditions [27].

A previous study reported the results of two-dimensional NMR profiling of the *S. cerevisiae* ESR to a variety of stressors; 36 metabolites were detected with differential resonance intensities dependent on stress condition [28]. Of that subset, 15 were differential in our data when one or more stress conditions (calcium, CsA, FK506) were compared to the wild-type (Supplementary Table S4). Succinic acid, for example, showed a reverse correlation to this study with significantly higher expression in calcium, CsA and FK506 datasets when compared to the wild-type.

### 3.3. MS/MS Spectral Library Identification

**Figure 3:**
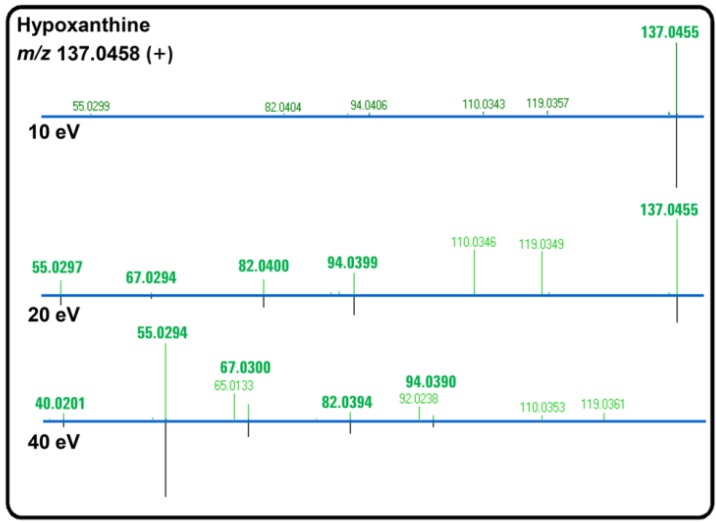
The MS/MS identified compound, hypoxanthine, is shown at three different collision cell energies (10, 20 and 40 eV). Difference plots show acquired data on top with METLIN Personal Compound Database and Library (PCDL) spectra on the bottom; ion matches are highlighted. At the lowest collision cell energy of 10eV, only the parent ion signal is observed. This is replaced by a fragment ion signal at 20 and 40 eV, with no parent ion signal observed at 40 eV.

All the acquired MS/MS spectra were matched to an MS/MS spectral library representing over 2,000 chemical standards. This process resulted in confirming the identification of 57 metabolites (Supplementary Table S5). For example, hypoxanthine (*m*/*z* 137.0458) was detected by both untargeted and targeted data mining. Targeted mining results (based on empirical formula) for hypoxanthine revealed that average abundances were significantly (*p* < 0.05) different between wild-type/CsA and calcium/FK506 treatment conditions. Moreover, the purine biosynthesis and salvage pathway was relatively enriched with metabolites compared to other pathways. Figure 3 shows MS/MS spectral difference plots for hypoxanthine at collision energies of 10, 20 and 40 eV. Each difference plot contains the acquired MS/MS spectra above, with the matched library spectra for the standard below. Furthermore, a compelling pattern of increased signal in response to calcium treatment compared to wild-type for hypoxanthine was observed in MS data, while prior treatment with FK506 or CsA appeared to suppress hypoxanthine signal to levels lower than that observed in the wild-type (Supplementary Table S4). This could indicate a connection between calcineurin and hypoxanthine levels during homeostasis, as immunosuppressant drug treatment appeared to reduce the levels irrespective of subsequent calcium treatment.

### 3.4. Pathway Analysis

Of the 147 *S. cerevisiae* specific pathways curated in YeastCyc, 100 pathways were enriched with one or more metabolite matches to our annotated targeted data mining feature list. While there was redundancy across pathways, there were compelling links between some of the pathway enrichment results and the known yeast ESR.

**Figure 4:**
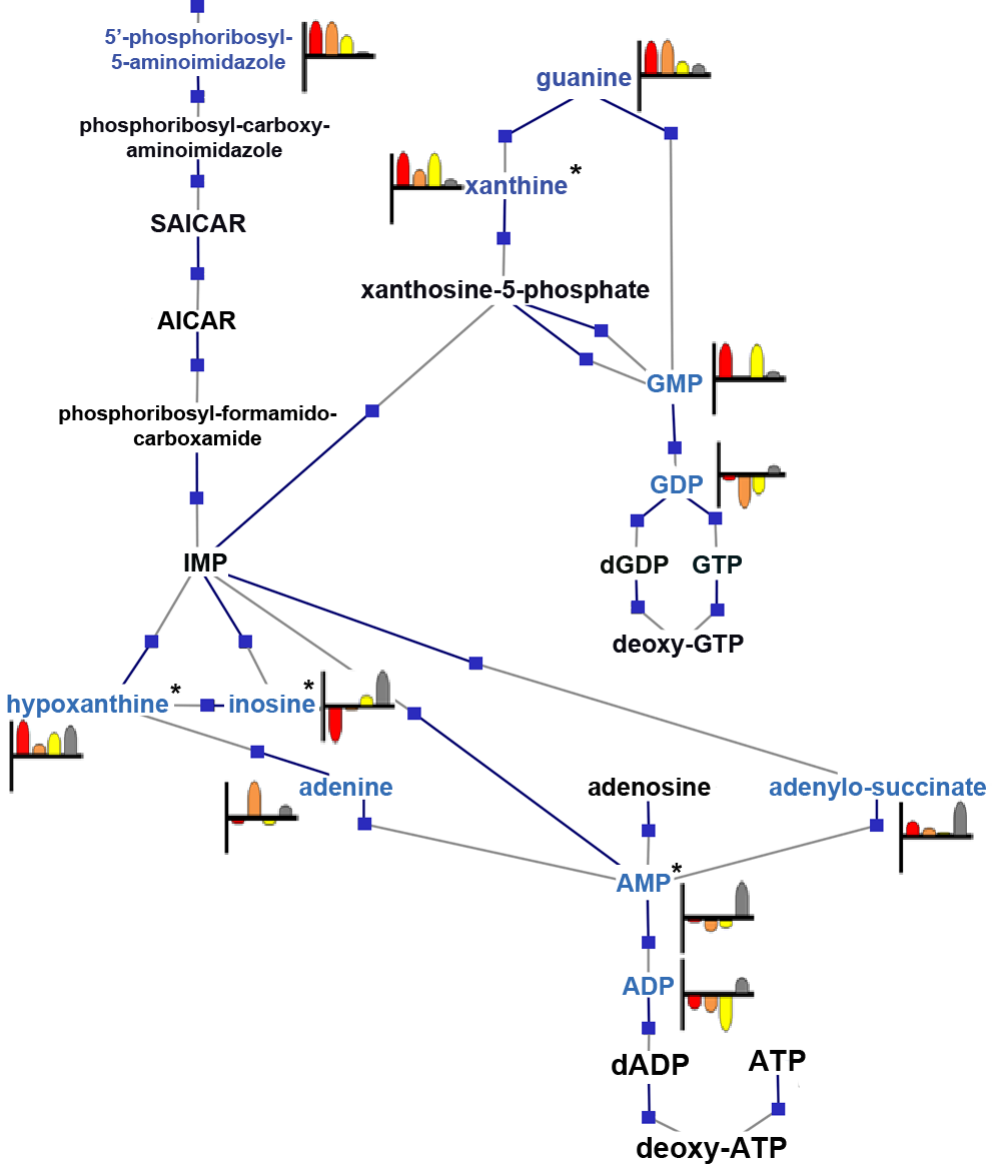
A portion of the purine biosynthesis and salvage pathway rendered from YeastCyc and enriched with the annotated feature and MS/MS (*) identified compound results. Metabolites detected are highlighted in blue and accompanied by a heat strip that shows log2 normalized relative abundance as detected per treatment condition; from left to right: calcium, red; CsA, orange; FK506, yellow; wild-type, grey. Boxes indicate gene/enzyme interaction nodes, which contain the genes described, but are not depicted.

The purine biosynthesis and salvage pathways in YeastCyc contains multiple genes reported to be involved in the yeast response to high glucose or fermentative conditions [16,29,30], as well as alkaline stress [31]. Our results revealed that 16 of the 40 annotated compounds in this pathway could be found at levels significant at *p* < 0.05 in our data, a portion of which is displayed in Figure 4.

Inosine abundance levels were the highest in the wild-type and the lowest in calcium treated samples, indicating that calcium stress had a significant suppressive effect on inosine levels, while CsA and FK506 pre-treatment attenuated this effect. Within this pathway, a purine nucleoside phosphorylase encoded by *PNP1* converts inosine to hypoxanthine and operates in conjunction with Hpt1p and Isn1p to modulate inosine levels, due to changing ATP, ADP and AMP concentrations in response to environmental conditions. *Hpt1* mutants have been reported to accumulate inosine when challenged by immediate increases in glucose concentration, indicating that they possess a modified stress response mechanism [30]. Considering the established role of Hpt1p in the conversion of inosine to hypoxanthine, it is also compelling that the highest hypoxanthine levels were observed in the calcium dataset with CsA and FK506 datasets, showing levels lower than the wild-type.

The sulfur amino acid biosynthesis pathway contains eight metabolites that we were able to be detected (Table 1). The levels of the metabolite, O-acetyl-L-homoserine, were lower in calcium and FK506 treatment conditions when compared to the wild-type. O-acetyl-L-homoserine is catalyzed by Met17p, a protein whose levels have been shown to be suppressed by exposure to the biomass conversion inhibitor, furfural [32]. This effect is reported for multiple enzymes that modulate the sulfur amino acid biosynthesis pathway and concurs with the enrichment of this pathway by our results, which further implicate it as a component of the *S. cerevisiae* ESR.

**Table 1:** Metabolites enriched to the sulfur amino acid biosynthesis pathway. The corrected *p*-value (*p* (Corr)), molecular formula and Kyoto Encyclopedia of Genes and Genomes (KEGG) ID is indicated. ND, not detected. Treatment conditions: WT, wild-type; CA, calcium treated only; CY, CsA followed by calcium treated; FK, FK506 followed by calcium treated.

Compound	*p* (Corr)	CA/WT	CY/WT	FK/WT	Molecular Formula	KEGG ID
L-cystathionine	2.11 × 10^−^^11^	−1.62	−1.35	−1.63	C_7_H_14_N_2_O_4_S	C02291
L-methionine	9.15 × 10^−17^	1.27	1.05	−1.13	C_5_H_11_NO2S	C00073
L-serine	2.47 × 10^−19^	−0.43	−0.30	−3.04	C_3_H_7_NO_3_	C00065
ADP	4.53 × 10^−14^	−0.87	−1.07	−2.32	C_10_H_15_N_5_O_10_P_2_	C00008
O-acetyl-L-homoserine	1.08 × 10^−4^	0.17	ND	0.09	C_6_H_11_NO_4_	C01077
L-aspartate-semialdehyde	1.40 × 10^−4^	−8.22	−10.54	−14.42	C_4_H_7_NO_3_	C00441
L-aspartate	7.23 × 10^−9^	−3.95	−0.23	1.83	C_4_H_7_NO_4_	C00049
L-glutamate	3.49 × 10^−6^	−2.69	1.98	1.93	C_5_H_9_NO_4_	C00025
CoA	1.01 × 10^−2^	2.42	3.85	3.75	C_21_H_36_N_7_O_16_P_3_S	C00010
acetyl-CoA	0	−10.76	−10.76	−8.84	C_23_H_38_N_7_O_17_P_3_S	C00024

**Figure 5:**
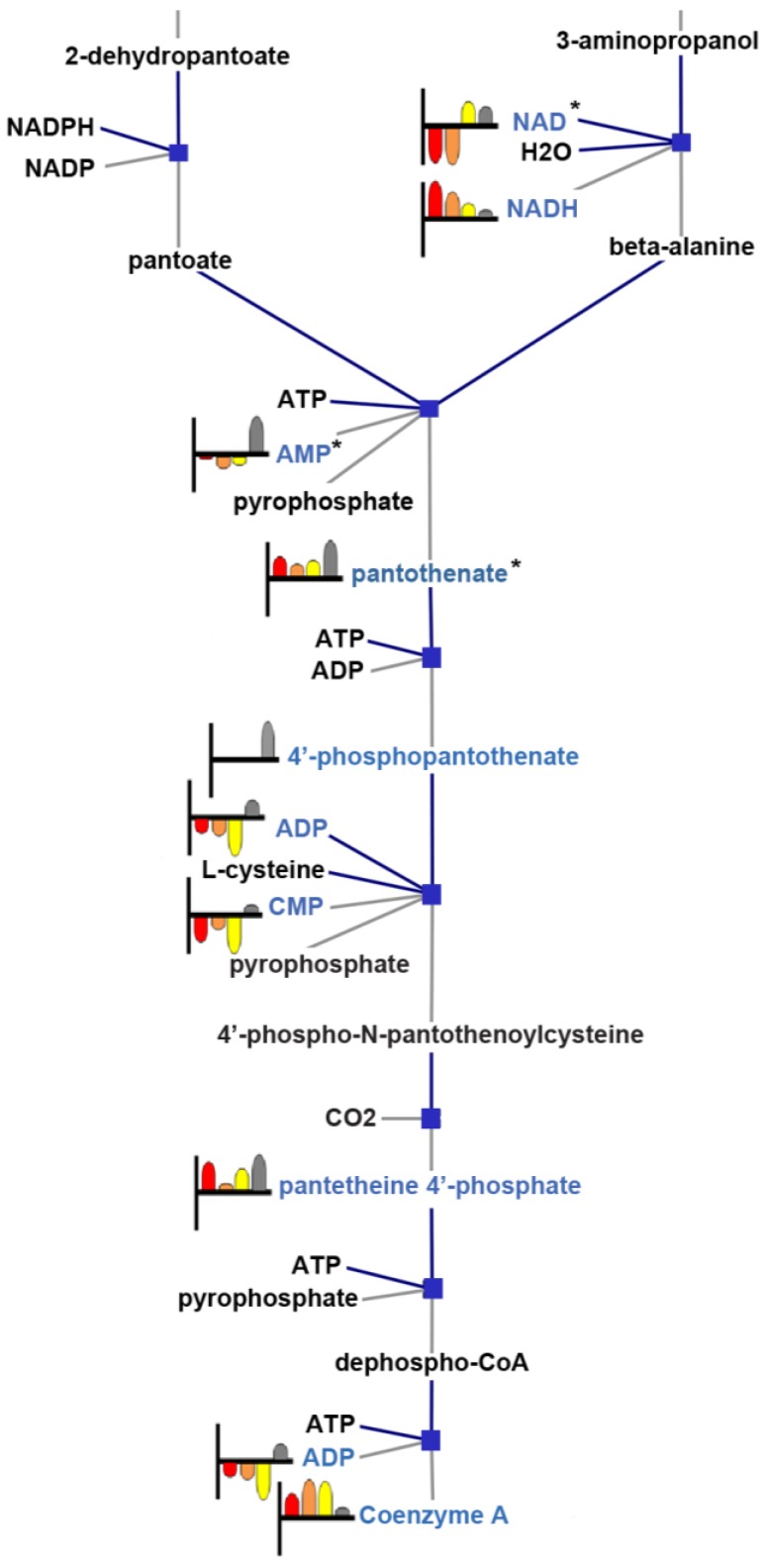
A portion of the pantothenate and coenzyme A biosynthesis pathway rendered from YeastCyc and enriched with the annotated features and MS/MS (*) identified compound results. The metabolites detected are highlighted inblue and accompanied by a heat strip that shows log2 normalized relative abundance, as detected per treatment condition; from left to right: calcium, red; CsA, orange; FK506, yellow; wild-type, grey. Boxes indicate gene/enzyme interaction nodes, which represent the genes described, but not depicted.

The pantothenate and coenzyme A biosynthesis pathway contains 29 annotated compounds, of which 12 were detected to be differential between at least two treatment conditions. A portion of this pathway containing our results is represented in Figure 5. Within this pathway are genes directly linked to the *S. cerevisiae* ESR, such as *FMS1*, reported by microarray analysis as a calcineurin-dependent gene responsive to calcium stress [3]. In addition, the regulatory subunit of Ppz1p encoded by *SIS2* (also known as *HAL3*) affects multiple regulatory functions, including the dephosphorylation and nuclear translocation of Crz1p, required for the transcriptional changes that affect the calcineurin response [33,34]. Upstream of *SIS2* within the pantothenate and coenzyme A biosynthesis pathway are the metabolites, pantothenate and cytidine monophosphate (CMP); downstream are pantetheine 4′-phosphate and coenzyme A (CoA). All of these metabolites are enriched as significant at *p* < 0.05 in our results and, except for CoA, were detected at the highest levels in the wild-type, an indication of the suppressive effects of the culture treatments monitored in our study.

BioCyc (and the YeastCyc content described) does not currently contain data or pathway relationships for genes implicated in the *S. cerevisiae* calcineurin-mediated stress response, such as *NRG1*, *ENA1*, *RIM101*, *PMC1* and *PMR1* [11,31,35], as well as the well-understood calcineurin activated transcription factor, *CRZ1* [2]. As the available content for pathway analysis and biological interpretation expands, it will be interesting to re-visit this pathway inference analysis approach in order to determine if there are specific correlations between the metabolite pools and gene expression in response to calcineurin-mediated calcium stress. Furthermore, interpretation of global, untargeted metabolomics results ideally should be considered in light of important factors, such as the complexity and interconnectivity of metabolic networks, as demonstrated by published studies on the growth response of *S. cerevisiae* to deletion mutants [36], exposure to high pH [37] and the scope of the calcineurin mediation of the calcium stress response [3,11], which makes any biological interpretation challenging.

## 4. Conclusions

Environmental perturbations are typically characterized by quick adjustment of cellular physiology. To demonstrate the utility of our technological and bioinformatic workflow, we compared metabolomic changes in response to calcium stress and two immunosuppressive drugs, FK506 and CsA. Features that were differentially abundant between pairwise conditions were annotated to or identified as compounds associated with the *S. cerevisiae* ESR. For example, hexadecanoic and octadecanoic fatty acid levels were reduced in response to all treatment or stress conditions in comparison to the wild-type. MS/MS-identified metabolites, such as inosine and hypoxanthine, showed relative abundances that were compelling when the established ESR role of the enzyme, Hpt1p, to which these metabolites are mechanistically linked, is considered. Annotated metabolites were projected onto curated biochemical pathways, suggesting that the purine biosynthesis and salvage pathway, sulfur amino acid biosynthesis and pantothenate and coenzyme A biosynthesis pathways were active in response to the calcium-induced stress response in our experiment. Our workflow showed promising results for global profiling using a dual data mining approach: targeted and untargeted, with pathway enrichment analysis results that can facilitate biological interpretation, detect unknown metabolites, understand metabolic pathway relationships, inform experimental design for targeted metabolomics or targeted proteomics and/or implicate which genes to analyze by microarray or PCR.

## Supplementary Materials

Supplementary materials can be accessed at: http://www.mdpi.com/2218-1989/3/4/1102/s1.

Supplementary File 1 Supplementary Materials (PDF, 253 KB)
