# Supplementary material for: Global LC/MS Metabolomics Profiling of Calcium Stressed and Immunosuppressant Drug Treated Saccharomyces cerevisiae
Source: Metabolites. 2013 Dec 6;3(4):1102–17. doi: 10.3390/metabo3041102 (PMC3937837; doi:10.3390/metabo3041102)
Supplement: Supplementary File 1 — Supplementary Materials (PDF, 253 KB) [file metabolites-03-01102-s001.pdf]

Supplementary Materials

N1. Yeast Culturing

Experimental conditions were examined (data not shown) to test parameters of the quenching and extraction methodology. Using untargeted data mining feature results to evaluate experimental parameters tested, we were able to incrementally increase signal and reproducibility to a point where the optimized protocol could be used for stress comparison study, the results of which are reported.

S. cerevisiae strain BJ5459 (generously supplied by Dr. Martha Cyert of Stanford University) was cultured at 30 °C in 0.8 L of YPD media (2% peptone, 2% dextrose, 1% yeast extract; MP Biomedical, Santa Ana, CA, USA) until an OD_{600} of 0.8 was measured using an Agilent 8,453 spectrophotometer (Agilent Technologies, Santa Clara, CA, USA), at which point cultures were challenged by control, calcium or calcium and drug exposure, resulting in four discrete culture types, referred to as "treatment conditions". *Wild type control culture (wild type)*: 4 mL of ET (90:10 ethanol:Tween 20) was added to culture at OD_{600} = 0.8; cultivation for 1 h; addition of 200 mL of YPD media; cultivation 15 min. *Calcium control culture (calcium)*: 4 mL of ET was added to culture at OD_{600} = 0.8; cultivation for 1 h; addition of 0.2 L of YPD media containing 1M $CaCl_2$ (final concentration, 0.2 M $CaCl_2$). *Drug (FK506) challenge followed by Ca^{2+} exposure (FK506)*: 4 mL of ET containing 1 mg/mL FK506 (final concentration, 5 µg/mL) were added to culture at OD_{600} = 0.8; cultivation for 1 h; addition of 0.2 L of YPD media containing 1 M $CaCl_2$ (final concentration, 0.2 M $CaCl_2$); cultivation 15 min. *Drug (Cyclosporin A) challenge followed by Ca^{2+} exposure (CsA)*: 4 mL of ET containing 1 mg/mL Cyclosporin A (final concentration, 5 µg/mL) were added to culture at OD_{600} = 0.8; cultivation for 1 h; addition of 0.2 L of YPD media containing 1 M $CaCl_2$ (final concentration, 0.2 M $CaCl_2$); cultivation 15 min.

After a 15-min exposure to 0.2 M $CaCl_2$ or media only control, cultures were centrifuged at 3,000 rpm for 5 min at 4 °C (Avanti J25i, Beckman Coulter, Brea, CA, USA), the supernatant discarded, the pellet washed with 0.2 L of 1x phosphate buffered saline (PBS), centrifuged, washed and centrifuged again. Previous unreported experiments showed a strong MS signal correlation between final data and a YPD media only control, an indication that residual media was being carried through the extraction process and contributing to the final signal. Therefore, two subsequent PBS washes were introduced in order to: (1) remove residual media prior to extraction in an isotonic solution that would prevent cell lysis and minimize metabolite extraction; and (2) remove extracellular metabolites and enable a more accurate endometabolomic measurement.

The pellet was then suspended in 1 mL of PBS, to which was added 2 mL of 4:1 methanol:PBS (pre-chilled in an isopropanol/dry ice bath) as a quenching solution, with previously described quenching techniques [1,2] used as a template. Tubes were then placed in an isopropanol/dry ice bath for 15 min, after which they were transferred to a lyophilizer (Virtis 3.5L DBT, SP Industries, Warminster, PA, USA) pre-chilled to −60 °C, dried and stored at −80 °C, pending extraction. In order to account for metabolites extracted by the cold methanol quench, a concern previously reported [1,3], tubes containing the quench solution and yeast pellet, with a total volume of less than 10 mL, were placed directly into the lyophilizer. Metabolites extracted in the quenching process were thus retained and present in the final signal after subsequent extraction.

N2. Metabolite Extraction and Sample Resuspension

Tubes were placed in chilled milling canisters and milled at 30 Hz for 3 × 1 minute cycles using a mixer mill (Retsch MM301, Haan, Germany). An isopropanol/dry ice bath was used to chill the canisters between milling cycles for 2 min. All tubes were then micro-centrifuged (Eppendorf 5415R, Hamburg, Germany) at 4,100 rpm and 4 °C for 5 min for biphasic separation. A 0.5 mL fraction of the polar phase was aspirated to a separate tube, and 0.5 mL of 1:1 methanol:water was added to the extraction tube. The tubes were briefly vortexed. Centrifugation, separation and polar phase removal was done a second time. The remaining non-polar phase was then aspirated into a separate tube.

After lyophilization (Virtis 3.5L DBT, SP Industries, Warminster, PA, USA) at −60 °C, the polar phase samples were resuspended in 100 µL of 50:50 water:methanol with 0.2% formic acid for reversed phase (RP) separation or in 100 µL of 10 mM ammonium acetate in 50:50 water:methanol for aqueous normal phase (ANP) separation. The non-polar phase samples were re-suspended in 100 µL of 50:50 water:methanol with 0.2% formic acid for reverse phase (RP) separation only.

Table S1. MS and MS/MS conditions.

**MS Conditions**	
Ion mode	ESI− positive and negative, APCI− positive
Drying gas temperature	325 °C
Vaporizer temperature	350 °C
Drying gas flow	10 L/min ESI, 5 L/min APCI
Nebulizer pressure	45 psi
Capillary voltage	4,000 V ESI positive/negative ion mode<br>3,500 V APCI positive ion mode
Spectra acquisition rate	1.4 spectra/s
**MS/MS Conditions**	
Quad resolution	High resolution
Ion mode	Both positive and negative
Drying gas temperature	325 °C
Drying gas flow	9 L/min
Nebulizer pressure	35 psig
Capillary voltage	4,000 V (positive mode)/3,500 V (negative mode)
Fragmentor	200 V
Skimmer	65 V
OCT1RFVpp	750 V
Isolation width	~1.3 m/z
Reference Delivery	Isocratic pump with 100:1 splitter
Reference pump flow	1 mL/min for 10 µL/min to nebulizer
Reference ions	Positive mode: 121.050873 and 922.009798<br>Negative mode: 119.036320 and 966.000725
Instrument mass range	1,700 Da
Acquisition rate	3.35 spectra/s
TOF * spectra mass range	25 to 1,000 m/z
Collision energy (eV)	10, 20 and 40 eV
Data storage	Centroid
Threshold	100 (MS) and 5 (MS/MS)
Instrument mode	Extended Dynamic Range

* TOF: Time of Flight.

Table S2. Two-hundred four differentially detected ($p < 0.05$) untargeted data mining features were annotated to the METLIN metabolite database. The corrected *p*-value, *p* (Corr), and log2 normalized relative abundances are indicated. ND, not detected. Treatment conditions: WT, wild-type, not drug or calcium treated; CA, calcium treated only; CY, Cyclosporin A followed by calcium treated; FK, FK506 followed by calcium treated.

Compound	*p* (Corr)	CA/ WT	CY/ WT	FK/ WT	Molecular Formula	KEGG ID
(1R,6R)-6-hydroxy-2-succinylcyclohexa-2,4-diene-1-carboxylate	2.44×10^{-4}	−10.22	−11.94	−10.25	$C_{11}H_{12}O_6$	C05817
(1S,2R,4S)-(-)-bornyl acetate	1.51×10^{-7}	1.52	14.09	3.11	$C_{12}H_{20}O_2$	C09837
(3S)-3,6-diaminohexanoate	9.85×10^{-18}	−1.29	−1.64	−1.61	$C_6H_{14}N_2O_2$	C01142
(3S,4S)-3-hydroxytetradecane-1,3,4-tricarboxylic acid	1.86×10^{-2}	1.42	−5.52	−5.52	$C_{17}H_{30}O_7$	C04529
(Ac)2-L-Lys-D-Ala-D-Ala	4.78×10^{-12}	−11.37	−11.37	−11.37	$C_{16}H_{28}N_4O_6$	C03326
(R)-10-hydroxystearic acid	8.17×10^{-4}	−15.59	−13.58	−9.50	$C_{18}H_{36}O_3$	C03195
(R)-2-amino-3-hydroxypropanoic acid	2.23×10^{-19}	−0.43	−0.34	−1.13	$C_3H_7NO_3$	C00740
(R)-2-methylmalate	8.54×10^{-4}	1.69	10.64	ND	$C_5H_8O_5$	C02612
(R)-4-phosphopantoate	4.63×10^{-2}	−5.16	−5.16	−5.16	$C_6H_{13}O_7P$	C18911
(S)-ACPA	7.85×10^{-12}	−1.90	−0.88	−1.12	$C_8H_{10}N_2O_5$	C13673
(S)-ATPA	1.08×10^{-3}	1.61	9.32	ND	$C_{10}H_{16}N_2O_4$	C13733
(S)-mevalonic acid	1.25×10^{-3}	−3.83	−10.93	−12.73	$C_6H_{12}O_4$	C02104
(S)-N-[3-(3,4-methylenedioxyphenyl)-2-(acetylthio)methyl-1-oxoprolyl]-(S)-alanine benzyl ester	2.94×10^{-13}	−13.58	−13.58	−12.11	$C_{25}H_{28}N_2O_7S$	C01316
1,2,3,7,8,9-hexachlorodibenzofuran	2.69×10^{-3}	−9.94	−13.44	−9.73	$C_{12}H_2C_{l6}O$	C18108
1,4-beta-D-glucan	4.69×10^{-2}	10.11	8.08	6.68	$C_{18}H_{32}O_{18}$	C00760
12-(2,3-dihydroxycyclopentyl)-2-dodecanone	4.24×10^{-6}	−1.95	−12.25	−1.14	$C_{17}H_{32}O_3$	C14996
15-keto-PGF2alpha	4.15×10^{-3}	7.37	−1.48	−0.01	$C_{20}H_{32}O_5$	C05960
17beta-hydroxy-4-mercaptoandrost-4-en-3-one 4-acetate 17-propionate	4.15×10^{-2}	3.88	−3.54	−8.93	$C_{24}H_{34}O_4S$	C15180
17-ethynyl-5alpha-androstan-17beta-ol	1.18×10^{-4}	ND	8.76	ND	$C_{21}H_{32}O$	C15431
19-bromoaplysiatoxin	1.00×10^{-7}	−9.44	−9.44	−9.44	$C_{32}H_{46}Br_2O_{10}$	C16770
19-norpregna-4,17(20)-dien-3-one	1.17×10^{-5}	−11.11	−16.24	−11.09	$C_{20}H_{28}O$	C15016
1-aminocyclohexanecarboxylic acid	4.15×10^{-6}	ND	10.90	ND	$C_7H_{13}NO_2$	
1-O-(1Z-Tetradecenyl)-2-(9Z-octadecenoyl)-sn-glycerol	1.18×10^{-4}	−10.32	−8.64	−10.32	$C_{35}H_{66}O_4$	C13864
1-Octen-3-ol-3-o-beta-D-xylopyranosyl (1-6)-beta-D-glucopyranoside	ND	ND	ND	13.01	$C_{19}H_{34}O_{10}$	C17614
1-O-hexadecyl-2-(9Z-octadecenoyl)-sn-glycerol	7.97×10^{-4}	−7.93	−11.16	1.83	$C_{37}H_{72}O_4$	C13862
1-oxa-2-oxo-3-methylcycloheptane	4.08×10^{-4}	−8.69	−8.69	−8.69	$C_7H_{12}O_2$	C10976
2-(3-carboxy-3-aminopropyl)-L-histidine	4.03×10^{-2}	−1.02	−2.11	−5.51	$C_{10}H_{16}N_4O_4$	C04441

Table S2. *Cont.*

Compound	*p* (Corr)	CA/ WT	CY/ WT	FK/ WT	Molecular Formula	KEGG ID
2,2-dimethyl-3-(4-methoxyphenyl)-4-ethyl-2H-1-benzopyran-7-ol acetate	3.25×10^{-4}	ND	9.21	1.47	$C_{22}H_{24}O_4$	C15055
2,3-dihydroxy-3-methylvaleric acid	1.18×10^{-4}	ND	9.24	ND	$C_6H_{12}O_4$	C04104
2,4,5-trichlorophenoxyacetic acid	3.43×10^{-3}	1.80	−10.24	−6.55	$C_8H_5Cl_3O_3$	C07100
26-hydroxybrassinolide	7.40×10^{-4}	6.93	ND	ND	$C_{28}H_{48}O_7$	C19874
2alpha,3alpha-(difluoromethylene)-5alpha-androstan-17beta-ol acetate	4.15×10^{-6}	−10.10	−10.10	−10.10	$C_{22}H_{32}F_2O_2$	C15322
2-anthramine	1.18×10^{-4}	ND	9.13	ND	$C_{14}H_{11}N$	C14417
2′-deoxyuridine	1.36×10^{-4}	−12.10	−12.09	−11.91	$C_9H_{12}N_2O_5$	C00526
2-furoic acid	4.94×10^{-2}	−4.86	−6.64	−6.64	$C_5H_4O_3$	C01546
2-mercaptoethanesulfonic acid	8.38×10^{-5}	15.56	8.34	10.04	$C_2H_6O_3S_2$	C03576
2-methyl-3-oxoadipate	1.18×10^{-4}	ND	8.41	ND	$C_7H_{10}O_5$	C18307
2-methylbutanal	1.90×10^{-3}	4.83	9.49	9.68	$C_5H_{10}O$	C02223
2-methylcitric acid	2.75×10^{-2}	−6.94	1.79	5.02	$C_7H_{10}O_7$	
2-methylthiobenzothiazole	1.73×10^{-3}	8.40	ND	1.69	$C_8H_7NS_2$	C10910
2-protocatechoylphloroglucinolcarboxylate	3.21×10^{-2}	1.90	13.59	7.74	$C_{14}H_{10}O_8$	C04524
3-(4-chlorophenyl)-2H-1-benzopyran-2-one	5.76×10^{-3}	−1.43	−12.20	−12.16	$C_{15}H_9ClO_2$	C15189
3-(Carboxycarbonylamino)-L-alanine	1.94×10^{-2}	−13.13	−13.13	−10.74	$C_5H_8N_2O_5$	C04209
3beta-fluoro-5alpha-androstan-17beta-ol	1.09×10^{-8}	−1.59	−13.60	−1.33	$C_{19}H_{31}FO$	C15330
3-dehydro-L-threonate	6.97×10^{-7}	−0.51	−1.07	−1.48	$C_4H_6O_5$	C03064
3-epihydroxymugineic acid	2.58×10^{-5}	9.30	ND	14.67	$C_{12}H_{20}N_2O_9$	C15501
3-fluoro-D-alanine	4.28×10^{-4}	9.02	ND	1.33	$C_3H_6FNO_2$	C02638
3-hydroxy-4-hydroxymethyl-2-methylpyridine-5-carboxylate	6.51×10^{-3}	−11.87	−6.88	−8.59	$C_8H_9NO_4$	C04773
3-hydroxyisoheptanoic acid	1.33×10^{-2}	3.05	−7.46	1.49	$C_7H_{14}O_3$	N/A
3-methyl-2-butenoic acid	6.49×10^{-4}	−4.29	−12.82	−6.02	$C_5H_8O_2$	N/A
3-tert-butyl-5-methylcatechol	1.18×10^{-4}	ND	8.38	ND	$C_{11}H_{16}O_2$	C03929
3′-UMP	5.94×10^{-3}	−6.64	−6.64	−6.64	$C9H_{13}N_2O_9P$	C01368
4,4′-diapophytoene	8.24×10^{-7}	−3.65	−13.92	−3.09	$C_{30}H_{48}$	C16144
4-[(hydroxymethyl)nitrosoamino]-1-(3-pyridinyl)-1-butanone	1.53×10^{-2}	−0.69	−0.59	−5.88	$C_{10}H_{13}N_3O_3$	C19563
4-[2-(5-carboxy-2-hydroxy-3-methoxyphenyl)-2-oxoethylidene]-2-hydroxy-2-pentenedioate	6.15×10^{-14}	−1.05	−1.50	−1.45	$C_{15}H_{12}O_{10}$	C18349
4-amino-1-piperidinecarboxylic acid	3.65×10^{-4}	−2.89	−12.97	−11.21	$C_6H_{12}N_2O_2$	C16837
4-aminophenylalanine	4.22×10^{-11}	−12.28	−17.30	−0.78	$C_9H_{12}N_2O_2$	C12033
4′-cinnamoylmussatioside	7.44×10^{-13}	11.71	13.06	12.99	$C_{34}H_{44}O_{16}$	C10439
4′-demethyldeoxypodophyllotoxin	4.08×10^{-4}	7.90	ND	ND	$C_{21}H_{20}O_7$	C10552
4-fluoro-L-threonine	1.57×10^{-7}	−1.21	−0.77	−0.84	$C_4H_8FNO_3$	C15533
4-guanidinobutanamide	1.18×10^{-4}	ND	9.06	ND	$C_5H_{12}N_4O$	C03078
4-heptyloxyphenol	1.13×10^{-5}	−8.86	−17.63	−19.85	$C_{13}H_{20}O_2$	C14236

Table S2. *Cont.*

Compound	*p* (Corr)	CA/ WT	CY/ WT	FK/ WT	Molecular Formula	KEGG ID
4-hexyloxyphenol	4.64×10^{-8}	0.19	1.53	0.54	$C_{12}H_{18}O_2$	C14305
4-hydroxycinnamyl aldehyde	1.06×10^{-6}	−7.04	−15.30	−13.69	$C_9H_8O_2$	C05608
4-hydroxyglucobrassicin	2.28×10^{-12}	−12.81	−12.81	−12.81	$C_{16}H_{20}N_2O_{10}S_2$	C08422
4′-hydroxyropivacaine	6.84×10^{-3}	5.76	13.79	1.88	$C_{17}H_{26}N_2O_2$	C16574
4-methylaminobutyrate	1.18×10^{-4}	ND	8.92	ND	$C_5H_{11}NO_2$	C15987
4-methylimidazole	2.79×10^{-2}	−12.36	−14.42	−10.20	$C_4H_6N_2$	C19262
4-methylthiobutylthiohydroximate	3.63×10^{-3}	9.12	ND	3.32	$C_5H_{11}NOS_2$	C17243
4-n-hexylphenol	1.71×10^{-2}	8.73	2.35	8.88	$C_{12}H_{18}O$	C14465
4-octenedioic acid	4.05×10^{-3}	−8.84	−8.76	−12.25	$C_8H_{12}O_4$	
4-PIOL	4.06×10^{-4}	−3.38	−10.98	−0.66	$C_8H_{12}N_2O_2$	C13710
4Z,7Z,10Z-octadecatrienenitrile	0	−16.27	−16.27	−16.27	$C_{18}H_{29}N$	C13832
5-(3-methyltriazen-1-yl)imidazole-4-carboxamide	6.91×10^{-4}	8.65	11.35	10.10	$C_5H_8N_6O$	C16250
5,8-tetradecadienoic acid	8.26×10^{-4}	−1.51	8.80	−1.51	$C_{14}H_{24}O_2$	N/A
5alpha-cholesta-8-en-3-one	6.73×10^{-3}	ND	5.29	8.48	$C_{27}H_{44}O$	N/A
5-aminopentanamide	2.44×10^{-4}	−9.55	−14.50	−7.93	$C_5H_{12}N_2O$	C00990
5-carboxyvanillic acid	4.80×10^{-8}	12.44	ND	ND	$C_9H_8O_6$	C18338
5-coprostanol	2.04×10^{-3}	ND	1.87	8.89	$C_{27}H_{48}O$	N/A
5-dehydroepisterol	9.31×10^{-9}	−1.75	−14.11	−1.66	$C_{28}H_{44}O$	C15780
5-deoxy-5-aminoshikimic acid	3.65×10^{-3}	−8.23	−9.85	−8.27	$C_7H_{11}NO_4$	C12121
5′-guanylate diphosphate (guanosine diphosphate)	4.08×10^{-4}	−8.03	−8.03	−8.03	$C_{10}H_{15}N_5O_{11}P_2$	C00035
5-hydroxyindoleacetic acid	1.17×10^{-2}	−4.54	−4.58	−11.26	$C_{10}H_9NO_3$	C05635
6-(isopropylthio) purine	6.13×10^{-5}	0.69	1.12	1.28	$C_8H_{10}N_4S$	C15347
6-hydroxyl-1,6-dihydropurine ribonucleoside	5.95×10^{-4}	−13.42	−8.46	−13.48	$C_{10}H_{14}N_4O_5$	C04583
6-oxabicyclo[3.1.0]hexane-2-undecanoic acid methyl ester	1.59×10^{-3}	−8.26	−6.64	−8.26	$C_{17}H_{30}O_3$	C15465
8-methylnonenoate	3.25×10^{-6}	−11.60	−11.60	−9.97	$C_{10}H_{18}O_2$	N/A
8Z,11Z,14Z-heptadecatrienoic acid	1.18×10^{-4}	ND	8.90	ND	$C_{17}H_{28}O_2$	C16344
9-anthroic acid	3.48×10^{-4}	−0.45	−1.02	−0.72	$C_{15}H_{10}O_2$	C13699
Ac-Tyr-OEt	4.99×10^{-3}	1.74	9.68	1.59	$C_{13}H_{17}NO_4$	C01657
adenosine 3′-monophosphate	1.35×10^{-11}	−0.60	−1.03	−0.87	$C_{10}H_{14}N_5O_7P$	C01367
adipate semialdehyde	1.22×10^{-2}	−1.60	−8.12	−8.12	$C_6H_{10}O_3$	C06102
aethusin	6.24×10^{-3}	−9.36	−11.32	−5.69	$C_{13}H_{14}$	C08395
all-trans-hexaprenyl diphosphate	7.29×10^{-14}	−3.26	−3.26	17.51	$C_{30}H_{52}O_7P_2$	C01230
aloperine	2.14×10^{-3}	9.59	15.12	3.66	$C_{15}H_{24}N_2$	C10748
alpha-ergocryptine	1.99×10^{-8}	ND	ND	12.16	$C_{32}H_{41}N_5O_5$	C07545
americine	6.97×10^{-3}	ND	9.78	1.56	$C_{31}H_{39}N_5O_4$	C09996
aminoDHQ	8.38×10^{-4}	4.43	−14.74	−1.76	$C_7H_{11}NO_5$	C12109
argininic acid	1.11×10^{-2}	−1.33	−10.97	−7.74	$C_6H_{13}N_3O_3$	N/A
aspidoalbine	1.79×10^{-5}	5.66	2.70	13.45	$C_{24}H_{32}N_2O_5$	C09038
auriculine	2.10×10^{-41}	ND	ND	15.99	$C_{31}H_{45}NO_8$	C10280

Table S2. *Cont.*

Compound	*p* (Corr)	CA/ WT	CY/ WT	FK/ WT	Molecular Formula	KEGG ID
australine	1.94×10^{-2}	−9.15	−9.15	−9.15	$C_8H_{15}NO_4$	C10132
benthiavalicarb isopropyl	3.29×10^{-2}	10.33	13.46	6.87	$C_{18}H_{24}FN_3O_3S$	C18415
benzo[g]chrysene	1.37×10^{-2}	−5.32	−7.04	3.65	$C_{22}H_{14}$	C19340
benzyl 2-methyl-3-oxobutanoate	9.39×10^{-4}	1.46	ND	8.89	$C_{12}H_{14}O_3$	C04000
benzyl isothiocyanate	1.18×10^{-4}	ND	ND	8.31	C_8H_7NS	C03098
beta-zearalanol	2.18×10^{-2}	−8.49	−8.49	−8.49	$C_{18}H_{26}O_5$	C14753
B-norcholest-4-en-3-one	8.24×10^{-5}	−1.73	−12.57	−2.96	$C_{26}H_{42}O$	C15119
broussonin C	1.18×10^{-4}	ND	8.55	ND	$C_{20}H_{24}O_3$	C09524
burseran	7.90×10^{-6}	−1.34	9.60	8.33	$C_{22}H_{26}O_6$	C10547
capsi-amide	1.46×10^{-3}	8.35	ND	8.35	$C_{17}H_{35}NO$	C17515
caulophylline	2.80×10^{-7}	ND	12.37	ND	$C_{12}H_{16}N_2O$	C10760
chikusetsusaponin III	0	ND	ND	15.29	$C_{47}H_{80}O_{17}$	C17539
chimyl alcohol	9.31×10^{-4}	ND	5.17	10.17	$C_{19}H_{40}O_3$	C13859
cholestane	2.26×10^{-6}	−13.89	−17.40	−8.66	$C_{27}H_{48}$	C19661
coenzyme A (CoA)	9.29×10^{-3}	8.12	ND	ND	$C_{21}H_{36}N_7O_{16}P_3S$	C00010
Compound III(S)	2.18×10^{-2}	ND	ND	7.73	$C_{22}H_{30}N_2O_3$	C06495
cucurbitacin E	4.78×10^{-12}	ND	ND	11.13	$C_{32}H_{44}O_8$	C08797
cymarin	7.60×10^{-5}	−4.30	−8.48	−12.56	$C_{30}H_{44}O_9$	C08859
dehydroabietic acid	8.25×10^{-14}	−13.47	−15.17	−15.17	$C_{20}H_{28}O_2$	C12078
dehydrocurdione	1.18×10^{-4}	ND	8.72	ND	$C_{15}H_{22}O_2$	C16949
di(2-ethylhexyl) adipate	1.05×10^{-5}	14.41	−4.33	9.67	$C_{22}H_{42}O_4$	C14240
dibenzthion	2.48×10^{-2}	1.51	8.86	5.97	$C_{17}H_{18}N_2S_2$	C12767
dihydroflavonol	1.54×10^{-3}	ND	1.59	8.17	$C_{15}H_{12}O_3$	C15570
dihydrophloroglucinol	1.32×10^{-3}	−2.52	−10.85	−10.61	$C_6H_8O_3$	C06719
D-proline	3.60×10^{-3}	−7.64	−12.70	−5.83	$C_5H_9NO_2$	C16435
ergosta-5,7,22,24(28)-tetraen-3beta-ol	1.40×10^{-5}	−4.66	−13.28	−1.39	$C_{28}H_{42}O$	C05440
estra-1,3,5(10)-triene-3,6alpha,17beta-triol triacetate	1.99×10^{-3}	−11.36	−13.35	−1.78	$C_{24}H_{30}O_6$	C15382
euphorbia factor Ti2	5.88×10^{-10}	10.27	13.10	14.01	$C_{32}H_{42}O_7$	C09091
flaccidin B	9.29×10^{-3}	−5.41	−5.41	−5.41	$C_{41}H_{64}O_{12}$	C08943
flavine mononucleotide (FMN)	3.20×10^{-6}	5.41	14.49	16.24	$C_{17}H_{21}N_4O_9P$	C00061
flumioxazin	1.06×10^{-4}	−1.65	−12.72	−5.06	$C_{19}H_{15}FN_2O_4$	C11035
germine ×	4.45×10^{-5}	−2.60	−6.99	−12.36	$C_{27}H_{43}NO_8$	C10807
gibberellin A5	2.12×10^{-2}	−8.08	−10.02	−10.02	$C_{19}H_{22}O_5$	C11871
glutaric acid	1.03×10^{-4}	10.32	12.50	11.75	$C_5H_8O_4$	C00489
glycidyl oleate	5.47×10^{-6}	0.85	−10.36	1.08	$C_{21}H_{38}O_3$	C19426
gonane	9.46×10^{-5}	−12.61	−14.26	−9.33	$C_{17}H_{28}$	C19639
gymnemic acid I	0	−14.93	−14.93	4.76	$C_{43}H_{66}O_{14}$	C08947
halfordinol	0	−16.05	−16.05	−16.05	$C_{14}H_{10}N_2O_2$	C10596
heptadecane	1.18×10^{-4}	ND	10.68	ND	$C_{17}H_{36}$	C01816
histidylleucine	1.18×10^{-4}	ND	10.17	ND	$C_{12}H_{20}N_4O_3$	C05010
inuline	0	ND	ND	16.45	$C_{32}H_{46}N_2O_8$	C08659
iridotrial glucoside	1.39×10^{-4}	−2.09	−2.09	13.16	$C_{16}H_{24}O_8$	C11653

Table S2. *Cont.*

Compound	*p* (Corr)	CA/ WT	CY/ WT	FK/ WT	Molecular Formula	KEGG ID
jodrellin A	1.65×10^{-4}	−11.46	−13.35	−15.27	$C_{24}H_{32}O_8$	C09121
kikkanol C	1.18×10^{-4}	ND	9.16	ND	$C_{15}H_{24}O_3$	C17605
kuraridinol	5.17×10^{-13}	1.56	1.91	2.01	$C_{26}H_{32}O_7$	C17445
kynurenine	9.29×10^{-3}	5.77	ND	ND	$C_{10}H_{12}N_2O_3$	C00328
leucyl-leucyl-norleucine	0	−12.54	−12.54	−12.54	$C_{18}H_{35}N_3O_4$	C11328
lignoceric acid	2.28×10^{-2}	4.21	9.93	4.27	$C_{24}H_{48}O_2$	C08320
lipoic acid	0	−17.41	−17.41	−17.41	$C_8H_{14}O_2S_2$	C00725
L-isoleucyl-L-proline	5.04×10^{-7}	11.41	10.34	13.07	$C_{11}H_{20}N_2O_3$	N/A
loganin	2.83×10^{-7}	10.88	9.15	12.25	$C_{17}H_{26}O_{10}$	C01433
mammeisin	7.31×10^{-3}	ND	ND	9.43	$C_{25}H_{26}O_5$	C09275
mannopine	1.08×10^{-3}	−10.08	−10.08	−10.08	$C_{11}H_{22}N_2O_8$	C16692
methyl N-(a-methylbutyryl)glycine	2.92×10^{-8}	1.10	0.90	0.45	$C_8H_{15}NO_3$	N/A
methyl propenyl ketone	9.29×10^{-3}	−5.97	−5.97	−5.97	C_5H_8O	N/A
morroniside	6.82×10^{-4}	−9.34	7.44	2.29	$C_{17}H_{26}O_{11}$	C17000
N-(6-aminohexanoyl)-6-aminohexanoic acid	1.16×10^{-8}	−2.18	−0.89	−11.98	$C_{12}H_{24}N_2O_3$	C01255
N,N′-diphenyl-p-phenylenediamine	9.01×10^{-3}	−1.60	−10.80	−7.17	$C_{18}H_{16}N_2$	C14501
N5-(L-1-Carboxyethyl)-L-ornithine	1.18×10^{-4}	ND	9.93	ND	$C_8H_{16}N_2O_4$	C04210
N6-(delta2-Isopentenyl)-adenosine 5′-monophosphate	2.18×10^{-2}	−7.31	−9.10	−9.10	$C_{15}H_{22}N_5O_7P$	C04713
N-acetyldemethylphosphinothricin tripeptide	1.89×10^{-2}	5.61	6.94	ND	$C_{12}H_{22}N_3O_7P$	C17950
N-acetylneuraminic acid	6.91×10^{-3}	ND	12.85	7.43	$C_{11}H_{19}NO_9$	C00270
N-glycolyl-D-mannosaminolactone	9.64×10^{-4}	7.09	7.04	7.12	$C_8H_{13}NO_7$	C03948
nigakilactone F	2.73×10^{-2}	5.27	12.37	3.49	$C_{22}H_{32}O_7$	C17031
N-methylpelletierine	1.67×10^{-6}	5.26	ND	14.40	$C_9H_{17}NO$	C06184
oleandrose	8.35×10^{-5}	1.46	10.41	ND	$C_7H_{14}O_4$	C08237
ophiopogonin A	4.15×10^{-6}	ND	ND	10.09	$C_{41}H_{64}O_{13}$	C17041
ophiopogonin B	0	ND	ND	18.57	$C_{39}H_{62}O_{12}$	C17038
oxyacanthine	9.29×10^{-3}	5.68	7.25	7.59	$C_{37}H_{40}N_2O_6$	C09598
p-coumaroyl quinic acid	2.69×10^{-3}	14.20	8.77	10.55	$C_{16}H_{18}O_8$	C12208
pentahydroxyflavanone	2.64×10^{-2}	−4.14	0.51	0.71	$C_{15}H_{12}O_7$	C05911
pheneturide	0	−15.63	−15.63	−15.63	$C_{11}H_{14}N_2O_2$	C12590
phenylacetylglycine dimethylamide	1.94×10^{-2}	−8.96	−8.96	−8.96	$C_{12}H_{16}N_2O_2$	C12958
phytosphingosine	1.05×10^{-3}	−12.17	−8.78	−8.79	$C_{18}H_{39}NO_3$	C12144
pilocarpidine	0	−16.88	−16.88	−0.58	$C_{10}H_{14}N_2O_2$	C17964
pirimicarb	4.15×10^{-6}	−10.32	−15.39	−2.15	$C_{11}H_{18}N_4O_2$	C11079
presqualene diphosphate	4.15×10^{-6}	ND	ND	10.50	$C_{30}H_{52}O_7P_2$	C03428
protoporphyrin IX	7.31×10^{-3}	ND	ND	9.64	$C_{34}H_{36}N_4O_4$	C02191
purine	1.18×10^{-4}	ND	ND	9.89	$C_5H_4N_4$	C15587
pyridoxal phosphate	4.32×10^{-5}	−9.02	−15.95	−3.55	$C_8H_{10}NO_6P$	C00018
pyriminobac-methyl	2.22×10^{-11}	1.35	0.97	0.90	$C_{17}H_{19}N_3O_6$	C18486
retronecine	1.18×10^{-4}	ND	9.37	ND	$C_8H_{13}NO_2$	C06177

Table S2. *Cont.*

Compound	*p* (Corr)	CA/ WT	CY/ WT	FK/ WT	Molecular Formula	KEGG ID
rhizocticin D	1.79×10^{-4}	1.36	11.12	6.88	$C_{17}H_{33}N_6O_7P$	C17961
rubrophen	1.61×10^{-3}	ND	8.98	ND	$C_{22}H_{20}O_6$	C14612
S-adenosylhomocysteine	2.13×10^{-4}	1.09	0.51	0.65	$C_{14}H_{20}N_6O_5S$	C00021
S-nitroso-L-glutathione	3.54×10^{-4}	11.77	2.85	2.85	$C_{10}H_{16}N_4O_7S$	N/A
sphinganine	6.26×10^{-4}	−12.11	−10.37	−5.40	$C_{18}H_{39}NO_2$	C00836
spinosyn D	0	ND	ND	18.09	$C_{42}H_{67}NO_{10}$	C11056
succinic anhydride	5.70×10^{-26}	1.99	2.26	1.51	$C_4H_4O_3$	C19524
succinoadenosine	7.31×10^{-3}	9.29	16.40	8.99	$C_{14}H_{17}N_5O_8$	N/A
sugeonyl acetate	1.18×10^{-4}	ND	9.21	ND	$C_{17}H_{24}O_3$	C17506
tenulin	1.61×10^{-3}	−13.08	−13.08	−13.08	$C_{17}H_{22}O_5$	C09557
tetradecan1-ol	1.35×10^{-3}	1.84	9.89	ND	$C_{14}H_{30}O$	N/A
threonate	4.15×10^{-4}	−10.82	−0.42	−0.01	$C_4H_8O_5$	C01620
timosaponin A-III	0	ND	ND	16.88	$C_{39}H_{64}O_{13}$	C17075
tris(butoxyethyl)phosphate	1.29×10^{-4}	9.16	ND	ND	$C_{18}H_{39}O_7P$	C14446
tuberonic acid glucoside	3.34×10^{-10}	ND	ND	15.89	$C_{18}H_{28}O_9$	C08558
undecan-2-one	1.18×10^{-4}	ND	9.30	ND	$C_{11}H_{22}O$	C01875
vasicinol	6.01×10^{-10}	−16.40	−16.40	−11.37	$C_{11}H_{12}N_2O_2$	C10743
Vitamin D4	8.62×10^{-4}	−11.60	−8.13	−1.35	$C_{28}H_{46}O$	C18192
WIN I(S)	2.18×10^{-2}	−7.39	−7.39	−7.39	$C_{21}H_{28}N_2O_3$	C06497
xanthosine	7.40×10^{-4}	7.45	ND	ND	$C_{10}H_{12}N_4O_6$	C01762
zearalenone	4.19×10^{-5}	−15.14	−12.97	−15.14	$C_{18}H_{22}O_5$	C09981

Table S3. One-hundred eighty-eight non-redundant molecular formulas matched to differentially detected ($p < 0.05$) untargeted data mining results. The corrected *p*-value, *p* (Corr), and log2 normalized relative abundances are indicated. ND, not detected. Treatment conditions: WT, wild-type, not drug or calcium treated; CA, calcium treated only; CY, Cyclosporin A followed by calcium treated; FK, FK506 followed by calcium treated. MFG, Molecular Formula Generator algorithm.

Molecular Formula	*p* (Corr)	CA/WT	CY/WT	FK/WT	MFG Score
$C_3H_7NO_2S$	1.18×10^{-4}	ND	9.06	ND	82
$C_3H_7NOP_2$	3.16×10^{-4}	−8.68	−10.31	−10.31	84
$C_3H_9N_3S$	4.87×10^{-9}	6.38	19.02	18.53	83
$C_3H_9O_2PS_4$	8.03×10^{-6}	12.07	ND	1.70	83
$C_{45}H_{75}NO_2$	2.09×10^{-2}	−7.46	−9.05	−5.41	97
$C_4H_{10}NOP$	1.77×10^{-9}	−6.95	−19.81	−19.81	87
$C_4H_{11}N_5O_4P_2$	2.04×10^{-3}	ND	3.54	10.33	85
C_4H_8	2.18×10^{-2}	−1.84	7.57	−1.84	83
$C_4H_8N_4O_2S_2$	3.83×10^{-2}	6.64	ND	6.11	86
$C_4H_9NO_2S$	0	−16.04	−16.04	−16.04	85
$C_5H_{10}N_3O_7P$	7.88×10^{-4}	−0.53	−4.20	−11.43	86
$C_5H_{11}N_2O_2P$	9.28×10^{-5}	1.67	11.50	ND	83
$C_5H_{13}NO$	1.18×10^{-4}	ND	1.80	10.61	87

Table S3. *Cont.*

Molecular Formula	*p* (Corr)	CA/WT	CY/WT	FK/WT	MFG Score
$C_5H_{14}N_3P$	1.22×10^{-4}	ND	10.84	ND	80
$C_5H_7N_3S$	1.99×10^{-2}	−8.75	−8.75	−8.75	89
$C_6H_{13}N_8O_2P$	3.33×10^{-4}	−0.64	−4.94	−12.69	96
$C_6H_{13}O_{11}P_3S$	4.27×10^{-3}	−4.33	−5.92	−13.04	94
$C_6H_7N_2O_3PS_4$	5.94×10^{-3}	−7.18	−7.18	−7.18	90
$C_7H_{10}NO_8P$	2.79×10^{-3}	−9.80	−13.22	−11.36	80
$C_7H_{12}O_4P_2$	4.15×10^{-4}	−8.69	−8.69	−8.69	81
$C_7H_{13}N_2O_{17}P_3S$	5.90×10^{-6}	−2.82	−0.51	−12.84	92
$C_7H_{14}N_3OP$	1.18×10^{-4}	ND	8.47	ND	90
$C_7H_{14}N_3P$	9.54×10^{-4}	1.83	11.02	ND	92
$C_7H_{14}N_7O_2P_3$	2.48×10^{-5}	−11.59	−13.21	−10.03	85
$C7H17N_3OS_2$	1.18×10^{-4}	ND	9.30	ND	95
$C_7H_{18}N_4O_5P_2S$	4.65×10^{-2}	−5.06	−5.06	−5.06	94
$C_7H_{19}N_2P$	1.18×10^{-4}	ND	9.48	ND	83
$C_7H_5NO_2$	1.18×10^{-4}	ND	9.18	ND	84
$C_7H_5NO_3S_2$	3.80×10^{-2}	−0.02	−4.61	−0.44	93
$C_7H_5NOS_2$	7.19×10^{-7}	13.44	−1.66	3.42	98
$C_7H_6N_4O$	1.18×10^{-4}	ND	8.66	ND	80
$C_8H_{10}O_3S$	7.00×10^{-3}	2.08	−9.74	−1.69	88
$C_8H_{11}N_9O_4S$	4.59×10^{-2}	−8.69	−17.21	−6.38	94
$C_8H_{14}N_5O_2P$	1.17×10^{-2}	10.07	1.66	3.33	84
$C_8H_{16}N_4$	8.23×10^{-4}	1.46	9.32	ND	81
$C_8H_{18}O_2S$	1.18×10^{-4}	ND	10.15	ND	86
$C_8H_{22}N_2O_2P_2S_4$	3.49×10^{-3}	−8.57	−8.59	−11.87	92
$C_8H_{27}N_8O_3P$	6.99×10^{-13}	−15.51	−15.51	−13.73	83
$C_8H_7N_2OP_3$	9.29×10^{-3}	ND	7.46	1.48	80
C_9H16	1.38×10^{-4}	−10.85	−14.29	−7.66	95
$C_9H_{18}N_2O$	1.94×10^{-2}	8.13	7.19	12.62	98
$C_9H_{26}N_6S_2$	0	−0.10	−15.16	2.54	86
$C_9H_5N_3O_2$	1.77×10^{-9}	−0.76	−13.09	−19.27	85
$C_9H_8O_{13}P_2$	3.71×10^{-2}	−6.16	−2.88	−11.61	80
$C_{10}H_{15}N_2P$	1.11×10^{-3}	1.76	10.12	ND	90
$C_{10}H_{24}N_3O_2P_3$	6.50×10^{-5}	8.11	−3.19	−4.66	86
$C_{10}H_5N_5O_8$	7.63×10^{-4}	−4.31	−0.83	−11.30	81
$C_{11}H_{10}N_4OS_3$	4.10×10^{-2}	−4.79	−6.31	−6.31	88
$C_{11}H_{12}$	9.48×10^{-5}	−12.21	−13.96	−9.02	86
$C_{11}H_{12}N_6O$	4.80×10^{-8}	−11.21	−11.21	−11.21	86
$C_{11}H_{16}N_6$	2.28×10^{-12}	−13.67	−13.67	−13.67	85
$C_{11}H_{18}N_2O_2$	1.62×10^{-7}	−15.67	−14.00	−1.35	96
$C_{11}H_{25}N_2O_5P_3S$	2.36×10^{-2}	−8.28	−8.28	−4.57	86
$C_{11}H_{26}N_2O_2$	1.18×10^{-4}	ND	9.18	ND	81
$C_{11}H_{26}N_4O_4$	7.70×10^{-4}	ND	10.56	1.61	94
$C_{12}H_{14}$	4.15×10^{-6}	−9.77	−9.77	−9.77	82
$C_{12}H_{17}N_5O$	6.62×10^{-8}	−11.36	−14.41	−12.90	84

Table S3. *Cont.*

Molecular Formula	*p* (Corr)	CA/WT	CY/WT	FK/WT	MFG Score
$C_{12}H_{17}O_5PS_3$	4.32×10^{-8}	−0.93	−0.90	−1.16	91
$C_{12}H_{25}N_5O_3$	4.08×10^{-4}	−8.14	−8.14	−8.14	81
$C_{12}H_{26}N_{17}O_2P_3$	4.08×10^{-4}	7.48	ND	ND	83
$C_{12}H_{27}N_2O_2P$	1.67×10^{-6}	−2.60	−2.67	−2.77	99
$C_{12}H_8P_2$	5.94×10^{-3}	−6.44	−6.44	−6.44	83
$C_{13}H_{18}N_2O_{13}P_2$	1.19×10^{-5}	−14.66	−11.47	−9.89	80
$C_{13}H_{21}OP$	1.67×10^{-15}	−6.17	−20.01	−3.52	83
$C_{13}H_{28}P_2$	3.05×10^{-2}	15.05	12.70	8.50	84
$C_{13}H_{30}N_4O_3$	1.18×10^{-4}	ND	10.26	ND	93
$C_{14}H_{10}N_7O_{10}P$	5.94×10^{-3}	−6.40	−6.40	−6.40	81
$C_{14}H_{18}O_2$	4.70×10^{-5}	−14.23	−14.23	−14.23	80
$C_{14}H_{22}$	8.41×10^{-15}	−16.34	−14.66	−16.34	89
$C_{14}H_{25}PS$	1.18×10^{-4}	ND	10.29	ND	99
$C_{14}H_{32}OP_2$	7.39×10^{-4}	1.51	10.13	ND	97
$C_{14}H_{34}P_2$	1.18×10^{-4}	ND	10.76	ND	91
$C_{15}H_{10}N_2O$	1.99×10^{-8}	−12.38	−12.38	−12.38	87
$C_{15}H_{14}N_2$	0	−16.22	−16.22	−16.22	81
$C_{15}H_{18}N_8O_3$	1.06×10^{-5}	−9.66	−9.66	−9.66	85
$C_{15}H_{20}N_5O_{11}P$	1.24×10^{-3}	1.08	1.13	0.78	99
$C_{15}H_{27}NOS$	1.18×10^{-4}	ND	9.90	ND	88
$C_{15}H_{28}O_3$	2.23×10^{-6}	−2.09	−12.33	−1.30	85
$C_{15}H_{34}N_4O$	1.22×10^{-4}	ND	10.08	ND	83
$C_{15}H_{34}P_2$	1.18×10^{-4}	ND	10.94	ND	97
$C_{15}H_{39}N_4O_2P_3S$	0	ND	ND	17.71	84
$C_{16}H_{13}N_2O_4P_3$	5.94×10^{-3}	−6.29	−6.29	−6.29	83
$C_{16}H_{21}O_9PS_2$	5.08×10^{-5}	9.90	−3.73	11.33	87
$C_{16}H_{27}N_{22}OP$	0	−14.58	−14.58	−14.58	83
$C_{16}H_{37}N_{14}PS_2$	1.50×10^{-11}	ND	ND	15.46	91
$C_{16}H_{37}N_6O_5PS_2$	2.25×10^{-6}	9.25	9.87	9.83	85
$C_{16}H_9N_6O_{14}P$	1.94×10^{-2}	−8.65	−8.65	−8.65	81
$C_{17}H_{22}P_2$	1.18×10^{-4}	ND	9.19	ND	80
$C_{17}H_{27}N_2PS$	0	ND	ND	16.18	92
$C_{17}H_{34}N_3PS$	1.50×10^{-11}	ND	ND	15.38	85
$C_{17}H_{37}NO_2$	0	−15.17	−15.17	−15.17	94
$C_{17}H_{39}N_8O_7P$	0	ND	ND	20.42	99
$C_{17}H_{43}N_8O_7P$	0	ND	ND	19.50	99
$C_{18}H_{22}N_4OS$	1.18×10^{-4}	ND	8.41	ND	84
$C_{18}H_{26}NP$	2.04×10^{-3}	ND	3.32	9.68	81
$C_{18}H_{27}N_5O_2$	7.05×10^{-4}	8.82	1.46	ND	92
$C_{18}H_{32}N_2O_2$	7.07×10^{-4}	1.59	10.83	ND	96
$C_{18}H_{32}N_4O_5$	5.93×10^{-6}	−1.71	−0.72	−10.88	92
$C_{18}H_{32}NPS$	3.01×10^{-2}	0.44	−4.81	−9.73	91
$C_{18}H_{38}N_{12}S_2$	2.35×10^{-2}	−6.73	−4.04	−6.73	82
$C_{19}H_{24}$	1.49×10^{-3}	−3.17	−12.02	−4.74	86

Table S3. *Cont.*

Molecular Formula	p (Corr)	CA/WT	CY/WT	FK/WT	MFG Score
$C_{19}H_{38}O_{10}P_2$	1.73×10^{-4}	5.71	11.01	11.09	89
$C_{19}H_{40}$	8.71×10^{-4}	2.02	12.62	ND	92
$C_{19}H_{42}N_2O_2S$	1.20×10^{-6}	−12.20	−13.93	−12.26	88
$C_{19}H_{43}N_8O_8P$	0	ND	ND	21.04	99
$C_{19}H_{45}N_5OS_4$	4.78×10^{-4}	ND	9.34	1.65	80
$C_{19}H_{47}N_8O_8P$	0	ND	ND	19.77	99
$C_{20}H_{35}N_6O_8P_3S_2$	3.29×10^{-3}	−13.33	−6.79	−6.66	95
$C_{20}H_{36}N_{10}S_2$	0	ND	ND	16.29	91
$C_{20}H_{38}O_{11}$	9.52×10^{-27}	ND	ND	18.46	98
$C_{20}H_{40}N_4OP_2$	7.51×10^{-4}	1.57	10.46	ND	98
$C_{21}H_{21}N_{14}O_7PS$	5.94×10^{-3}	−6.47	−6.47	−6.47	98
$C_{21}H_{25}N_7$	9.29×10^{-3}	5.30	−1.31	−1.31	85
$C_{21}H_{28}N_{15}O_5P_3$	1.28×10^{-8}	12.29	11.86	12.33	80
$C_{21}H_{28}N_7O_{17}P_3$	0	−15.46	−15.46	−15.46	99
$C_{21}H_{32}N_7O_{13}P_3S_2$	2.37×10^{-3}	−0.76	−0.87	−7.61	96
$C_{21}H_{33}N_8P_3$	4.07×10^{-4}	ND	ND	12.22	82
$C_{21}H_{38}N_{10}O_7$	7.15×10^{-13}	ND	ND	14.63	98
$C_{21}H_{42}N_{22}O$	7.40×10^{-4}	6.80	ND	ND	80
$C_{21}H_{45}N_{11}O_7$	4.22×10^{-6}	ND	ND	10.05	97
$C_{21}H_{51}N_8O_9P$	0	ND	ND	19.81	99
$C_{22}H_{40}O_{13}$	0	ND	ND	12.73	89
$C_{22}H_{42}N_2O_{12}S_2$	4.43×10^{-3}	−10.73	−10.86	−10.81	92
$C_{22}H_{42}O_{12}$	7.15×10^{-13}	ND	ND	13.70	98
$C_{22}H_{42}O_{13}$	0	ND	ND	12.97	93
$C_{22}H_{45}NO_3$	0	ND	ND	14.85	81
$C_{22}H_{50}N_{12}O_2$	2.18×10^{-2}	−9.14	−9.14	−7.33	81
$C_{23}H_{38}O_3S_2$	5.15×10^{-4}	0.90	1.04	1.13	85
$C_{23}H_{41}N_4O_6PS$	0	13.27	13.42	13.30	95
$C_{23}H_{44}N_4O_8P_2S$	8.92×10^{-4}	2.75	9.64	9.77	97
$C_{23}H_{45}N_{11}O_8$	1.08×10^{-41}	ND	ND	16.36	98
$C_{23}H_{45}O_{12}P$	8.62×10^{-10}	1.59	1.64	1.52	98
$C_{23}H_{55}N_8O_{10}P$	0	ND	ND	19.63	99
$C_{24}H_{19}N_{12}O_4P_3$	6.20×10^{-12}	−10.63	−10.63	−10.63	80
$C_{24}H_{30}N_6O_{11}P_2S$	0	ND	13.13	ND	88
$C_{24}H_{38}N_7P_3$	3.01×10^{-3}	2.80	ND	8.40	80
$C_{24}H_{42}O_{12}$	4.15×10^{-6}	ND	ND	10.12	90
$C_{24}H_{46}O_{13}$	0	ND	ND	16.82	98
$C_{25}H_{20}N_2O_2S_2$	4.52×10^{-3}	−4.99	−6.68	3.58	97
$C_{25}H_{34}OS_3$	1.74×10^{-3}	7.63	1.81	16.38	89
$C_{25}H_{39}N_5S_2$	3.29×10^{-2}	6.61	6.74	6.96	86
$C_{25}H_{40}O$	6.26×10^{-4}	1.65	5.20	12.03	80
$C_{25}H_{47}O_{12}P$	2.44×10^{-13}	2.77	2.53	2.91	98
$C_{25}H_{49}N_{11}O_9$	2.25×10^{-42}	ND	ND	16.52	98
$C_{26}H_{36}N_2O_4$	1.18×10^{-4}	ND	10.07	ND	97

Table S3. *Cont.*

Molecular Formula	p (Corr)	CA/WT	CY/WT	FK/WT	MFG Score
$C_{27}H_{42}N_2O_{11}$	1.35×10^{-2}	−0.67	6.41	6.88	96
$C_{27}H_{51}N_{12}O_2PS$	2.35×10^{-2}	4.03	−2.69	−2.69	88
$C_{27}H_{53}N_{11}O_{10}$	5.90×10^{-41}	ND	ND	16.32	99
$C_{27}H_{59}N_8O_{12}P$	0	ND	ND	18.15	98
$C_{28}H_{34}O$	2.65×10^{-6}	−10.37	−12.01	−12.01	81
$C_{28}H_{38}$	1.52×10^{-6}	−1.60	−12.63	−1.32	82
$C_{28}H_{40}$	1.64×10^{-6}	−1.81	−14.26	−1.47	82
$C_{28}H_{42}$	1.26×10^{-6}	−1.85	−16.26	−1.74	94
$C_{28}H_{44}$	1.33×10^{-6}	−0.45	−14.12	0.17	92
$C_{28}H_{48}O_6P_2$	4.15×10^{-6}	ND	ND	10.17	95
$C_{28}H_{50}O_{14}$	1.99×10^{-8}	ND	ND	12.30	92
$C_{28}H_{56}N_{10}O_5S$	1.57×10^{-4}	8.18	12.16	8.06	97
$C_{28}H_{61}N_2O_2P_3S_3$	8.36×10^{-3}	−0.04	−6.85	−6.85	92
$C_{29}H_{52}O_{21}$	1.03×10^{-9}	10.38	13.19	13.55	97
$C_{29}H_{54}N_{10}O11$	7.15×10^{-13}	ND	ND	15.40	98
$C_{29}H_{54}O_4$	2.57×10^{-6}	−2.20	−12.89	−1.32	98
$C_{29}H_{57}N_{11}O_{11}$	3.86×10^{-42}	ND	ND	15.83	98
$C_{30}H_{49}N_3P_2S_2$	9.65×10^{-8}	−9.51	−9.51	−9.51	92
$C_{30}H_{52}N_7O_2P_3$	2.24×10^{-5}	8.72	ND	ND	93
$C_{30}H_{54}O_{15}$	7.15×10^{-13}	ND	ND	14.02	92
$C_{30}H_{57}N_5O_9P_2S$	4.17×10^{-2}	2.91	2.90	−5.69	92
$C_{30}H_{58}O$	7.98×10^{-4}	ND	4.89	9.95	88
$C_{30}H_{62}O_{16}$	0	ND	ND	19.29	98
$C_{31}H_{49}N_3P_2S$	5.97×10^{-3}	6.97	−0.04	−1.38	83
$C_{31}H_{54}N_{17}PS$	1.84×10^{-6}	−2.94	−9.93	−12.65	89
$C_{31}H_{56}O_4$	2.68×10^{-6}	−1.60	−12.32	−0.90	98
$C_{31}H_{57}N_{16}OPS$	5.88×10^{-6}	−1.78	−0.72	−10.74	96
$C_{31}H_{58}O_4$	1.95×10^{-6}	−2.28	−13.37	−1.42	98
$C_{32}H_{45}O_{14}PS$	1.56×10^{-4}	7.30	−2.87	−2.87	89
$C_{32}H_{58}O_{16}$	0	ND	ND	15.70	93
$C_{32}H_{65}NO$	4.39×10^{-15}	14.59	−1.83	14.58	98
$C_{33}H_{60}O_4$	2.23×10^{-6}	−1.93	−12.82	−1.07	98
$C_{33}H_{62}O_4$	2.47×10^{-4}	−8.48	−13.50	−2.84	98
$C_{33}H_{67}NO$	2.69×10^{-2}	6.86	−1.64	3.38	95
$C_{34}H_{62}O_{17}$	4.15×10^{-6}	ND	ND	10.50	95
$C_{34}H_{66}O_2$	6.07×10^{-4}	−1.68	3.39	10.29	89
$C_{34}H_{69}NO$	5.66×10^{-15}	15.70	−1.98	15.62	98
$C_{35}H_{56}N_7O_7PS$	9.09×10^{-3}	5.62	−1.37	−1.37	96
$C_{35}H_{62}O_4$	1.95×10^{-6}	−1.63	−13.70	−0.86	98
$C_{35}H_{64}O_4$	1.84×10^{-6}	−2.12	−14.01	−1.03	98
$C_{37}H_{66}O_4$	1.33×10^{-6}	−1.72	−13.66	−0.84	96
$C_{37}H_{68}O_4$	8.69×10^{-7}	−2.02	−14.29	−0.96	97
$C_{38}H_{59}O_3P_3S$	7.40×10^{-4}	7.28	ND	ND	84

Table S4. One-hundred eighty-four non-redundant, differentially expressed entities were extracted by targeted data mining. The corrected *p*-value, *p* (Corr), and log2 normalized relative abundances are indicated. ND, not detected. Treatment conditions: WT, wild-type, not drug or calcium treated; CA, calcium treated only; CY, Cyclosporin A followed by calcium treated; FK, FK506 followed by calcium treated.

Compound	*p* (Corr)	CA/WT	CY/WT	FK/WT	Molecular Formula	KEGG ID
(2E,6E)-farnesol	2.35×10^{-5}	−3.14	−0.53	−0.32	$C_{15}H_{26}O$	C01126
(2R,3S)-3-isopropylmalate	1.12×10^{-5}	0.57	0.71	0.71	$C_7H_{12}O_5$	C04411
(3S,5S)-3,5-diaminohexanoate	2.84×10^{-16}	−0.04	−0.67	−0.51	$C_6H_{14}N_2O_2$	C01186
(9Z)-hexadecenoic acid	8.23×10^{-4}	0.04	−3.27	−1.69	$C_{16}H_{30}O_2$	C08362
(9Z)-octadecenoic acid	1.17×10^{-6}	−0.19	−0.07	−0.17	$C_{18}H_{34}O_2$	C00712
(R)-mevalonate	1.89×10^{-5}	−3.87	−6.17	−1.67	$C_6H_{12}O_4$	C00418
(S)-1-pyrroline-5-carboxylate	0	6.74	ND	4.99	$C_5H_7NO_2$	C03912
(S)-2-acetolactate	4.06×10^{-26}	3.72	3.99	3.23	$C_5H_8O_4$	C06010
(S)-dihydroorotate	0	−6.88	−3.62	−5.23	$C_5H_6N_2O_4$	C00337
(S)-lactate	5.22×10^{-6}	−6.57	−2.41	−1.03	$C_3H_6O_3$	C00186
(S)-malate	1.78×10^{-3}	−0.65	−0.78	−1.06	$C_4H_6O_5$	C00149
1-(beta D ribofuranosyl) nicotinamide	0	1.58	6.25	3.14	$C_{11}H_{15}N_2O_5$	C03150
1-aminocyclopropane-1-carboxylate	8.24×10^{-1}	0.05	−4.13	0.04	$C_4H_7NO_2$	C01234
1-hexadecanol	1.28×10^{-17}	−6.89	−12.43	−2.23	$C_{16}H_{34}O$	C00823
1H-imidazole-4-ethanamine	5.46×10^{-1}	0.05	1.64	4.77	$C_5H_9N_3$	C00388
1-pyrroline-4-hydroxy-2-carboxylate	2.03×10^{-2}	0.45	−0.23	−1.05	$C_5H_7NO_3$	C04282
2(alpha-D-mannosyl)-D-glycerate	5.67×10^{-30}	−1.03	−0.06	0.25	$C_9H_{16}O_9$	C11544
2-amino-5-oxohexanoate	5.46×10^{-1}	4.24	3.96	3.89	$C_6H_{11}NO_3$	C05825
2-dehydro-3-deoxy-D-fuconate	0	3.22	−0.05	−0.14	$C_6H_{10}O_5$	C06159
2-hexaprenyl-3-methyl-5-hydroxy-6-methoxy-1,4-benzoquinone	0	ND	ND	5.80	$C_{38}H_{56}O_4$	C05805
2-isopropylmaleate	2.26×10^{-6}	0.73	0.87	0.90	$C_7H_{10}O_4$	C02631
2-methylbutanal	0	ND	ND	4.68	$C_5H_{10}O$	C02223
2-methylmaleate	0	ND	−7.24	−5.43	$C_5H_6O_4$	C02226
2-oxoglutaramate	4.44×10^{-9}	−9.87	−0.80	−2.81	$C_5H_7NO_4$	C00940
2-succinyl-5-enolpyruvyl-6-hydroxy-3-cyclohexene-1-carboxylate	0	−11.63	−14.79	−14.79	$C_{14}H_{16}O_9$	C16519
3-(4-hydroxyphenyl)lactate	2.19×10^{-3}	7.35	5.78	7.34	$C_9H_{10}O_4$	C03672
3-hydroxyanthranilate	3.56×10^{-11}	10.07	9.12	2.09	$C_7H_7NO_3$	C00632
3-hydroxy-L-kynurenine	0	5.99	0.18	0.21	$C_{10}H_{12}N_2O_4$	C03227
3-methyl-2-oxobutanoate	0	−13.95	−9.10	−4.05	$C_5H_8O_3$	C00141
3-methylthiopropanal	0	−6.99	−6.99	4.65	$C_4H_8O_S$	N/A
3-phospho-D-glycerate	0	−3.76	−5.40	−1.60	$C_3H_7O_7P$	C00197
3-sulfolactate	0	−6.03	−4.27	−6.03	$C_3H_6O_6S$	C16069

Table S4. *Cont.*

Compound	*p* (Corr)	CA/WT	CY/WT	FK/WT	Molecular Formula	KEGG ID
3″-UMP	1.15×10^{-7}	−1.75	−3.80	−0.08	$C_9H_{13}N_2O_9P$	C01368
3-ureidopropionate	7.27×10^{-14}	−0.62	−0.71	−1.37	$C_4H_8N_2O_3$	C02642
4-aminobenzoate	0	5.51	2.58	−1.81	$C_7H_7NO_2$	C00568
4-aminobutyraldehyde	1.18×10^{-2}	4.57	−3.80	ND	C_4H_9NO	C00555
4-coumarate	0	−14.87	−4.97	−14.87	$C_9H_8O_3$	C00811
4-guanidinobutanal	0	−5.34	−5.34	−5.34	$C_5H_{11}N_3O$	C02647
4-guanidinobutanamide	1.08×10^{-4}	−9.29	−0.19	4.16	$C_5H_{12}N_4O$	C03078
4-hydroxyphenylpyruvate	6.36×10^{-4}	0.30	0.46	0.35	$C_9H_8O_4$	C01179
4-methyl-2-oxopentanoate	0	8.33	6.05	2.06	$C_6H_{10}O_3$	C00233
5 alpha-cholesta-7,24-dien-3beta-ol	0	10.34	ND	2.07	$C_{27}H_{44}O$	C05439
5-acetamidovalerate	1.93×10^{-9}	−0.02	15.54	15.64	$C_7H_{13}NO_3$	
5-amino-2-oxopentanoic acid	0	−5.49	−5.49	−5.49	$C_5H_9NO_3$	C01110
5-aminoimidazole	2.76×10^{-5}	0.32	2.25	0.35	$C_3H_5N_3$	C05239
5-aminopentanamide	9.67×10^{-3}	1.52	−2.57	−0.88	$C_5H_{12}N_2O$	C00990
5′-phosphoribosyl-5-aminoimidazole	0	12.84	12.68	7.26	$C_8H_{14}N_3O_7P$	C03373
5-phosphoribosyl-N-formylglycineamidine	2.96×10^{-4}	−2.92	0.75	−1.67	$C_8H_{16}N_3O_8P$	C04640
5-ureido-4-imidazole carboxylate	0	1.73	−5.23	−6.75	$C_5H_6N_4O_3$	C05515
7,8-dihydro-D-neopterin	0	ND	6.27	1.59	$C_9H_{13}N_5O_4$	C04874
7,8-dihydroneopterin 3′-phosphate	0	3.13	14.20	ND	$C_9H_{14}N_5O_7P$	C05925
acetyl-CoA	0	−10.76	−10.76	−8.84	$C_{23}H_{38}N_7O_{17}P_3S$	C00024
adenine	2.98×10^{-1}	−0.31	3.13	−0.35	$C_5H_5N_5$	D00034
adenylo-succinate	5.44×10^{-5}	0.43	0.22	0.07	$C_{14}H_{18}N_5O_{11}P$	C03794
ADP	4.53×10^{-14}	−0.87	−1.07	−2.32	$C_{10}H_{15}N_5O_{10}P_2$	C00008
ADP-ribose	0	4.61	ND	ND	$C_{15}H_{23}N_5O_{14}P_2$	C00301
all-trans-hexaprenyl diphosphate	0	ND	ND	17.40	$C_{30}H_{52}O_7P_2$	C01230
aminomethylpyrimidine	0	5.67	5.64	5.69	$C_6H_{10}N_4$	C20267
AMP	7.62×10^{-11}	−0.07	−0.32	−0.25	$C_{10}H_{14}N_5O_7P$	C00020
arbutin	0	1.83	12.30	7.00	$C_{12}H_{16}O_7$	C06186
carnosine	0	−15.62	−15.62	−15.62	$C_9H_{14}N_4O_3$	C00386
citrate	4.16×10^{-2}	4.26	3.85	4.55	$C_6H_8O_7$	C00158
CMP	0	−3.43	−1.82	−5.06	$C_9H_{14}N_3O_8P$	C05822
CoA	1.01×10^{-2}	2.42	3.85	3.75	$C_{21}H_{36}N_7O_{16}P_3S$	C00010
creatine	0	1.82	3.59	5.25	$C_4H_9N_3O_2$	C00300
cytidine	5.98×10^{-3}	−0.49	1.40	−0.60	$C_9H_{13}N_3O_5$	C00475
D-4′-phosphopantothenate	0	−16.10	−16.10	−16.10	$C_9H_{18}NO_8P$	C03492
D-altronate	7.62×10^{-11}	0.42	11.24	6.90	$C_6H_{12}O_7$	C00817
dAMP	1.98×10^{-4}	−3.22	−6.58	−0.13	$C_{10}H_{14}N_5O_6P$	C00360
decanoic acid	3.64×10^{-7}	−0.21	0.29	0.02	$C_{10}H_{20}O_2$	C01571
deoxyguanosine	6.15×10^{-1}	1.95	2.28	1.87	$C_{10}H_{13}N_5O_4$	C00330
deoxyribose	0	5.77	−1.90	16.23	$C_5H_{10}O_4$	C01801
deoxyshikonin	0	ND	5.32	1.74	$C_{16}H_{16}O_4$	C18133

Table S4. *Cont.*

Compound	*p* (Corr)	CA/WT	CY/WT	FK/WT	Molecular Formula	KEGG ID
D-fructose	1.61 × 10−9	0.08	0.85	1.41	$C_6H_{12}O_6$	C00095
D-glucono-1,5-lactone 6-phosphate	7.34 × 10−2	1.71	4.99	8.51	$C_6H_{11}O_9P$	C01236
D-glycerate	2.01×10^{-7}	4.10	3.60	1.91	$C_3H_6O_4$	C00258
dGTP	1.76×10^{-11}	2.34	−0.20	2.29	$C_{10}H_{16}N_5O_{13}P_3$	C00286
dihydroechinofuran	8.87×10^{-11}	0.19	0.23	0.32	$C_{16}H_{18}O_3$	C18134
dodecanoic acid	3.10×10^{-7}	0.01	0.33	0.06	$C_{12}H_{24}O_2$	C02679
D-ribose 5-phosphate	0	−6.87	−6.87	−6.87	$C_5H_{11}O_8P$	C00117
D-sorbitol	0	15.03	14.98	11.17	$C_6H_{14}O_6$	C00794
FMN	7.26×10^{-4}	−0.05	0.35	0.25	$C_{17}H_{21}N_4O_9P$	C00061
formylaminopyrimidine	3.92×10^{-2}	−0.34	−0.03	−1.53	$C_7H_{10}N_4O$	C19872
fumarate	0	−7.71	−7.71	−7.71	$C_4H_4O_4$	C00122
futalosine	2.13×10^{-6}	1.98	2.38	2.46	$C_{19}H_{18}N_4O_7$	C16999
gamma-glutamyl-gamma-aminobutyrate	0	1.82	−11.63	−8.44	$C_9H_{16}N_2O_5$	C15767
gamma-L-glutamylputrescine	0	−15.67	−15.67	−15.67	$C_9H_{19}N_3O_3$	C15699
gamma-tocopherol	1.38×10^{-2}	0.05	4.23	−6.15	$C_{28}H_{48}O_2$	C02483
GDP	1.07×10^{-8}	−0.51	−4.11	−2.29	$C_{10}H_{15}N_5O_{11}P_2$	C00035
geranyl-hydroxybenzoate	0	−8.56	−6.40	−10.67	$C_{17}H_{22}O_3$	C18131
glutathione	4.56×10^{-4}	2.31	13.73	9.13	$C_{10}H_{17}N_3O_6S$	C00051
glutathione disulfide	9.54×10^{-3}	0.07	1.82	1.87	$C_{20}H_{32}N_6O_{12}S_2$	C00127
glycerol	5.09×10^{-3}	−1.25	−0.41	−0.84	$C_3H_8O_3$	C00116
GMP	2.85×10^{-8}	5.57	0.03	5.26	$C_{10}H_{14}N_5O_8P$	C00144
guanidinoacetate	0	7.21	−8.82	7.07	$C_3H_7N_3O_2$	C00581
guanine	1.90×10^{-3}	3.25	3.32	1.24	$C_5H_5N_5O$	C00242
guanosine	2.27×10^{-3}	8.05	7.48	7.85	$C_{10}H_{13}N_5O_5$	C00387
guanosine 3″-diphosphate 5″-triphosphate	0	1.83	−7.55	−5.67	$C_{10}H_{18}N_5O_{20}P_5$	C04494
hexadecanoic acid	6.79×10^{-3}	0.25	0.41	0.28	$C_{16}H_{32}O_2$	C00249
homoisocitrate	1.22×10^{-15}	4.85	2.14	4.07	$C_7H_{10}O_7$	C05662
hypoxanthine	5.05×10^{-3}	1.16	0.36	0.75	$C_5H_4N_4O$	C00262
imidazol-5-yl-pyruvate	4.41×10^{-9}	−0.42	−3.83	−3.99	$C_6H_6N_2O_3$	C03277
indole-3-ethanol	0	10.69	6.22	15.33	$C_{10}H_{11}NO$	C00955
inosine	5.67×10^{-30}	−1.01	−0.04	0.27	$C_{10}H_{12}N_4O_5$	C00294
isobutanal	0	−7.91	−11.51	−11.51	C_4H_8O	N/A
L-2-aminoadipate adenylate	0	0.72	−12.50	−12.50	$C_{16}H_{23}N_6O_{10}P$	C05560
L-alanine	4.79×10^{-3}	−2.27	1.76	−0.35	$C_3H_7NO_2$	C00041
L-arginine	1.69×10^{-19}	−1.28	−1.48	−1.54	$C_6H_{14}N_4O_2$	C00062
L-aspartate	7.23×10^{-9}	−3.95	−0.23	1.83	$C_4H_7NO_4$	C00049
L-aspartate-semialdehyde	1.40×10^{-4}	−8.22	−10.54	−14.42	$C_4H_7NO_3$	C00441
L-citrulline	1.30×10^{-15}	0.46	−0.01	−0.08	$C_6H_{13}N_3O_3$	C00327
L-cystathionine	2.11×10^{-11}	−1.62	−1.35	−1.63	$C_7H_{14}N_2O_4S$	C02291
L-cystine	0	16.15	15.98	13.79	$C_6H_{12}N_2O_4S_2$	C00491
L-glutamate	3.49×10^{-6}	−2.69	1.98	1.93	$C_5H_9NO_4$	C00025

Table S4. *Cont.*

Compound	*p* (Corr)	CA/WT	CY/WT	FK/WT	Molecular Formula	KEGG ID
L-glutamyl 5-phosphate	0	6.61	ND	3.09	$C_5H_{10}NO_7P$	C03287
L-histidine	5.20×10^{-14}	−0.64	−1.15	−0.89	$C_6H_9N_3O_2$	C00135
LL-2,6-diaminoheptanedioate	4.13×10^{-4}	1.88	0.35	0.74	$C_7H_{14}N_2O_4$	C00666
L-lactaldehyde	0	2.00	2.02	5.96	$C_3H_6O_2$	C00424
L-leucine	0	−2.53	17.93	17.62	$C_6H_{13}NO_2$	C00123
L-methionine	9.15×10^{-17}	1.27	1.05	−1.13	$C_5H_{11}NO_2S$	C00073
L-methionine S-oxide	0	−9.63	−4.82	−11.43	$C_5H_{11}NO_3S$	C02989
L-ornithine	5.56×10^{-13}	−0.38	−1.04	−1.11	$C_5H_{12}N_2O_2$	C00077
L-phenylalanine	2.29×10^{-11}	−0.10	−0.17	−0.39	$C_9H_{11}NO_2$	C00079
L-pipecolate	6.39×10^{-3}	−0.42	0.97	1.17	$C_6H_{11}NO_2$	C00408
L-proline	0	ND	5.67	7.64	$C_5H_9NO_2$	C00148
L-saccharopine	7.62×10^{-11}	−0.07	−0.47	−0.21	$C_{11}H_{20}N_2O_6$	C00449
L-serine	2.47×10^{-19}	−0.43	−0.30	−3.04	$C_3H_7NO_3$	C00065
L-threonine	3.43×10^{-15}	1.51	1.35	−0.56	$C_4H_9NO_3$	C00188
L-tryptophan	2.50×10^{-3}	6.69	3.13	6.78	$C_{11}H_{12}N_2O_2$	C00078
L-tyrosine	0	ND	ND	6.73	$C_9H_{11}NO_3$	C00082
L-valine	0	−2.46	17.34	9.82	$C_5H_{11}NO_2$	C00183
L-xylonate	0	−14.71	−5.96	−2.36	$C_5H_{10}O_6$	C05411
mevalonate-5-phosphate	0	−10.30	−10.30	−6.98	$C_6H_{13}O_7P$	C01107
mevalonate-diphosphate	0	1.59	4.82	1.51	$C_6H_{14}O_{10}P_2$	C01143
N(pi)-methyl-L-histidine	0	−3.76	−3.97	−3.90	$C_7H_{11}N_3O_2$	C01152
N2-(D-1-carboxyethyl)-L-lysine	7.70×10^{-1}	3.26	−0.03	−1.64	$C_9H_{18}N_2O_4$	C04020
N2-acetyl-L-lysine	6.46×10^{-1}	2.06	−0.06	0.03	$C_8H_{16}N_2O_3$	C12989
N6-acetyl-N6-hydroxy-L-lysine	5.09×10^{-5}	1.35	0.65	0.61	$C_8H_{16}N_2O_4$	C03955
N-acetyl-D-glucosamine-6-phosphate	0	6.49	ND	ND	$C_8H_{16}NO_9P$	C00357
N-acetyl-L-glutamate	0	1.84	9.21	1.81	$C_7H_{11}NO_5$	C00624
N-acetyl-L-glutamate 5-semialdehyde	5.05×10^{-3}	4.13	1.60	−0.14	$C_7H_{11}NO_4$	C01250
N-acetyl-L-ornithine	0	6.25	8.66	1.56	$C_7H_{14}N_2O_3$	C00437
NAD+	4.57×10^{-6}	−2.01	−2.11	1.29	$C_{21}H_{28}N_7O_{14}P_2$	C00003
NADH	0	5.02	3.33	1.72	$C_{21}H_{29}N_7O_{14}P_2$	C00004
N-carbamoyl-L-aspartate	1.18×10^{-2}	−0.19	2.00	2.28	$C_5H_8N_2O_5$	C00438
N-formiminoglycine	7.36×10^{-2}	−0.31	−2.06	−0.18	$C_3H_6N_2O_2$	C02718
nicotinamide	2.04×10^{-2}	4.94	4.58	4.90	$C_6H_6N_2O$	C00153
N-succinyl-L-citrulline	0	8.89	6.94	6.79	$C_{10}H_{17}N_3O_6$	C18048
O-acetyl-L-homoserine	1.08×10^{-4}	0.17	ND	0.09	$C_6H_{11}NO_4$	C01077
octadecanoic acid	5.22×10^{-13}	0.27	0.41	0.30	$C_{18}H_{36}O_2$	C01530
octanoate	3.78×10^{-6}	0.29	0.69	0.47	$C_8H_{16}O_2$	C06423
O-phospho-L-homoserine	1.51×10^{-1}	−1.90	−1.63	0.09	$C_4H_{10}NO_6P$	C01102
O-phospho-L-serine	5.91×10^{-1}	4.08	1.38	5.36	$C_3H_8NO_6P$	C01005
orotate	5.62×10^{-13}	0.38	−0.30	−2.54	$C_5H_4N_2O_4$	C00295
orotidine 5′-phosphate	0	−13.92	−13.92	−13.92	$C_{10}H_{13}N_2O_{11}P$	C01103

Table S4. *Cont.*

Compound	*p* (Corr)	CA/WT	CY/WT	FK/WT	Molecular Formula	KEGG ID
orthophosphate	6.36×10^{-2}	−2.51	−0.32	−5.28	H_3O_4P	C00009
palmitaldehyde	0	8.76	6.55	2.18	$C_{16}H_{32}O$	C00517
pantetheine 4′-phosphate	4.18×10^{-6}	0.80	0.18	0.60	$C_{11}H_{23}N_2O_7PS$	C01134
pantothenate	1.11×10^{-4}	0.52	0.34	0.46	$C_9H_{17}NO_5$	C00864
phenylacetate	4.11×10^{-2}	−1.65	4.59	−3.17	$C_8H_8O_2$	C07086
phosphocholine	2.31×10^{-2}	7.35	7.58	7.34	$C_5H_{13}NO_4P$	N/A
phosphoenolpyruvate	0	−6.71	−6.71	−2.74	$C_3H_5O_6P$	C00074
piperideine	6.88×10^{-3}	4.13	0.61	1.70	C_5H_9N	C06181
protoporphyrinogen IX	1.12×10^{-1}	1.82	2.09	10.71	$C_{34}H_{40}N_4O_4$	C01079
putrescine	0	−5.29	−6.92	−5.15	$C_4H_{12}N_2$	C00134
pyrrole-2-carboxylate	0	−6.57	−4.39	−4.34	$C_5H_5NO_2$	C05942
S-(-)-ureidoglycolate	0	3.16	12.77	3.20	$C_3H_6N_2O_4$	C00603
S-adenosyl-L-homocysteine	8.85×10^{-10}	4.78	4.44	4.48	$C_{14}H_{20}N_6O_5S$	C00021
salicin	3.13×10^{-5}	4.35	10.58	12.67	$C_{13}H_{18}O_7$	C01451
sedoheptulose 7-phosphate	0	−3.87	−12.44	−12.43	$C_7H_{15}O_{10}P$	C05382
shikimate-3-phosphate	0	−13.01	−16.61	−16.61	$C_7H_{11}O_8P$	C03175
S-lactoyl-glutathione	0	ND	4.98	3.31	$C_{13}H_{21}N_3O_8S$	C03451
S-methyl-5′-thioadenosine	5.33×10^{-8}	0.27	0.39	−0.20	$C_{11}H_{15}N_5O_3S$	C00170
spermidine	0	2.19	13.38	8.76	$C_7H_{19}N_3$	C00315
S-ribosyl-L-homocysteine	0	−4.73	2.20	4.62	$C_9H_{17}NO_6S$	C03539
succinic acid	0	3.80	5.63	5.75	$C_4H_6O_4$	C00042
tetradecanoic acid	2.51×10^{-8}	0.31	0.51	0.33	$C_{14}H_{28}O_2$	C06424
tyrosol	0	−4.33	−6.53	−4.08	$C_8H_{10}O_2$	C06044
uracil	0	−7.47	−5.59	−7.47	$C_4H_4N_2O_2$	C00106
urate	5.00×10^{-5}	−2.48	−2.14	−0.49	$C_5H_4N_4O_3$	C00366
ureidoglycine	0	5.64	3.17	13.04	$C_3H_7N_3O_3$	C02091
uridine	0	−3.91	−7.74	−5.75	$C_9H_{12}N_2O_6$	C00299
urocanate	0	ND	8.18	ND	$C_6H_6N_2O_2$	C00785
xanthine	2.85×10^{-20}	5.16	2.65	5.05	$C_5H_4N_4O_2$	C00385
xanthosine	0	8.86	ND	ND	$C_{10}H_{12}N_4O_6$	C01762
xanthosine 5′-triphosphate	0	−0.16	−3.77	−5.50	$C_{10}H_{15}N_4O_{15}P_3$	C00700

Table S5. The identities of 57 compounds were confirmed by MS/MS. Fifty-seven compounds matched by acquired MS/MS spectra to an MS/MS spectral library by collision cell fragmentation energies (10, 20 and/or 40 eV).

Compound	Molecular Formula	KEGG ID
11-dehydro-thromboxane B2	$C_{20}H_{32}O_6$	C05964
2-hydroxyphenylacetate	$C_8H_8O_3$	C05852
3-(4-hydroxyphenyl)pyruvate	$C_9H_8O_4$	C01179
D-mannitol 1-phosphate	$C_6H_{15}O_9P$	C00644
1-methylxanthine	$C_6H_6N_4O_2$	C16358
2-aminoadipic acid	$C_6H_{11}NO_4$	C00956
3-hydroxy-3-methyl-glutaric acid	$C_6H_{10}O_5$	C03761
3-hydroxyanthranilic acid	$C_7H_7NO_3$	C00632
4-nitrophenol	$C_6H_5NO_3$	C00870
5′-methylthioadenosine	$C_{11}H_{15}N_5O_3S$	C00170
aconitic acid	$C_6H_6O_6$	C00417
adenosine	$C_{10}H_{13}N_5O_4$	C00212
adenosine 5′-diphosphate	$C_{10}H_{15}N_5O_{10}P_2$	C00008
adenosine monophosphate (AMP)	$C_{10}H_{14}N_5O_7P$	C00020
alpha-ketoglutarate	$C_5H_6O_5$	C00026
anthranilic acid	$C_7H_7NO_2$	C00108
benzoic acid	$C_7H_6O_2$	C00180
citric acid	$C_6H_8O_7$	C00158
deoxyadenosine monophosphate	$C_{10}H_{14}N_5O_6P$	C00360
deoxythymidine monophosphate (dTMP)	$C_{10}H_{15}N_2O_8P$	C00364
D-glycerate 3-phosphate	$C_3H_7O_7P$	C00197
embelin	$C_{17}H_{26}O_4$	C10342
flavin adenine dinucleotide (FAD)	$C_{27}H_{33}N_9O_{15}P_2$	C00016
glutamic Acid	$C_5H_9NO_4$	C00025
glutathione	$C_{10}H_{17}N_3O_6S$	C00051
glycerol 2-phosphate	$C_3H_9O_6P$	C02979
guanidylic acid (guanosine monophosphate)	$C_{10}H_{14}N_5O_8P$	C00144
hypoxanthine	$C_5H_4N_4O$	C00262
inosine	$C_{10}H_{12}N_4O_5$	C00294
itaconic acid	$C_5H_6O_4$	C00490
maleic acid	$C_4H_4O_4$	C01384
malic acid	$C_4H_6O_5$	C00149
methyl jasmonate	$C_{13}H_{20}O_3$	C11512
N6-(1,2-dicarboxyethyl)-AMP	$C_{14}H_{18}N_5O_{11}P$	C03794
N-acetyl-L-glutamic acid	$C_7H_{11}NO_5$	C00624
N-acetyl-L-phenylalanine	$C_{11}H_{13}NO_3$	C03519
N-formylanthranilic acid	$C_8H_7NO_3$	C05653
nicotinamide adenine dinucleotide (NAD)	$C_{21}H_{28}N_7O_{14}P_2$	C00003
oleic acid	$C_{18}H_{34}O_2$	C00712
orotic acid	$C_5H_4N_2O_4$	C00295
palmitic acid	$C_{16}H_{32}O_2$	C00249

Table S5. *Cont.*

Compound	Molecular Formula	KEGG ID
pantothenate	$C_9H_{17}NO_5$	C00864
phenylalanine	$C_9H_{11}NO_2$	C00079
propionic acid	$C_3H_6O_2$	C00163
pyroglutamic acid	$C_5H_7NO_3$	C01879
S-adenosyl-L-homocysteine	$C_{14}H_{20}N_6O_5S$	C00021
stearic acid	$C_{18}H_{36}O_2$	C01530
succinate	$C_4H_6O_4$	C00042
threonine	$C_4H_9NO_3$	C00188
trans-4-hydroxy-L-proline	$C_5H_9NO_3$	C01157
tryptophan	$C_{11}H_{12}N_2O_2$	C00078
uracil	$C_4H_4N_2O_2$	C00106
uridine	$C_9H_{12}N_2O_6$	C00299
uridine monophosphate (UMP)	$C_9H_{13}N_2O_9P$	C00105
urocanic acid	$C_6H_6N_2O_2$	C00785
xanthine	$C_5H_4N_4O_2$	C00385
xanthosine	$C_{10}H_{12}N_4O_6$	C01762

References

1. Villas-Boas, S.G.; Hojer-Pedersen, J.; Akesson, M.; Smedsgaard, J.; Nielsen, J. Global metabolite analysis of yeast: Evaluation of sample preparation methods. *Yeast* **2005**, *22*, 1155–1169.
2. Koning, W.; Dam, K. A method for the determination of changes of glycolytic metabolites in yeast on a subsecond time scale using extraction at neutral pH. *Anal. Biochem.* **1992**, *204*, 118–123.
3. Gonzalez, B.; Francois, J.; Renaud, M. A rapid and reliable method for metabolite extraction in yeast using boiling buffered ethanol. *Yeast* **1997**, *13*, 1347–1355.
